# Competing Effects of Network Architecture and Composition on Polydomain Liquid Crystal Elastomers

**DOI:** 10.1021/acs.macromol.5c02541

**Published:** 2026-01-22

**Authors:** David Taeyeun Yang, Callie W. Zheng, Chun Lam Clement Chan, Shawn M. Maguire, Emily C. Ostermann, Emily C. Davidson

**Affiliations:** Department of Chemical and Biological Engineering, 6740 Princeton University, Princeton, New Jersey 08544, United States

## Abstract

Main-chain liquid crystal elastomers (LCEs) are synthesized to investigate the interplay of the composition and network structure on LCE nematic-to-isotropic (N–I) transitions. We focus on networks synthesized from liquid crystalline oligomers reacted with tri- or tetrafunctional nonmesogenic cross-linker molecules. We find that coupling between mesogens and the polymer backbone increases with the degree of cross-linking. However, this enhanced coupling competes with mesogenic dilution arising from the cross-linker molecules to determine the N–I transition temperature (*T*_NI_). When cross-linker molecules are dilute, the degree of cross-linking directly correlates to the change in *T*_NI_ from the oligomer to LCE (Δ*T*_NI_) through mesogen–backbone coupling. In this regime, Δ*T*_NI_ ranges from 2.9 to 12.2 °C and 2.9–13.9 °C for tri- and tetrafunctional cross-linkers, respectively. At high cross-linker concentrations, deviations from this linear relationship appear. Further, the fractional mesogen content within an oligomer chain induces molecular weight-dependent mesogenic dilution effects arising from the flexible spacer molecules. Analysis of the N–I transition peak reveals a maximum latent heat per gram of mesogen (Δ*H*_NI,mes_) for this system.

## Graphical abstract

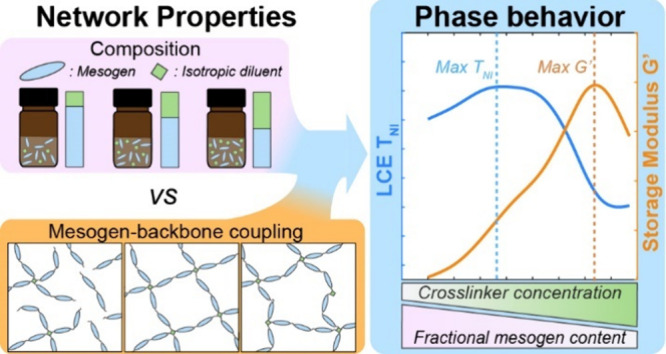

## Introduction

Liquid crystal elastomers (LCEs) are cross-linked polymer networks that exhibit anisotropic properties due to coupling between rod-shaped anisotropic liquid crystalline mesogens and the polymer backbone.^1,2^ Due to intermesogen van der Waals interactions and the minimization of excluded volume,^1,3^ the incorporation of mesogens into the network results in the formation of ordered liquid crystalline mesophases. Transitions between the liquid crystalline and isotropic phases can be induced through several stimuli, including heat,^1,2,4^ light,^5–8^ and mechanical strain.^9–11^ The most studied transition is the thermotropic phase transition between an ordered nematic (N) state and a disordered isotropic (I) state. When long-range alignment of the nematic director is programmed into the network structure through cross-linking, this N–I transition can be leveraged to induce a large macroscopic shape change^12–14^ due to the mechanical coupling of the mesogens with the polymer network and the associated transition between anisotropic and isotropic chain conformations.^15–17^ Additionally, within a certain strain regime, LCEs can be deformed at nearly constant stress as oriented domains rotate to align in the direction of strain. Ideal rotation of anisotropic chains incurs no free energy penalty, leading to this “soft elastic” behavior.^1,18,19^ This soft elastic plateau is theoretically sustained until the anisotropic chains are fully rotated, and the stress begins to induce chain stretching, causing conventional rubber elasticity to emerge.

The macroscopic shape change and soft elasticity of LCEs have been utilized for applications in soft robotics,^12,20–25^ sensor development,^26,27^ and energy dissipation.^28–30^ To this end, much work has gone into tailoring the temperature/breadth of their phase transitions, actuation strain, and soft elastic plateau by controlling the polymerization route and molecular composition of LCEs.^20,23,24,31–35^ In particular, the profound impacts of leveraging different cross-linking methods on the N–I transition can be understood through the divergence of LCE phase behavior from that of small-molecule LCs.

The thermotropic N–I transition for small-molecule LCs can be described by the Landau–de Gennes (LdG) theory. Here, the N–I transition is characterized by a discontinuous change in nematic order at a critical nematic-to-isotropic transition temperature, or *T*_NI_.^1,4^ While this transition is first-order, exhibiting a latent heat and discontinuity in volume, it is only weakly so, evidenced by pretransitional fluctuations implying its close proximity to a critical point at which it becomes second-order.^36^ Compared to small-molecule liquid crystals, an LCE’s N–I transition is observed over a broad temperature range, deviating from first-order behavior and limiting the speed and mechanical work capacity of actuation. It is theorized that these limitations are caused by quenched random disorder within the network due to “field disorder” imposed from cross-link junctions and “bond disorder” in the form of topological defects.^37–39^ An additional term in the LdG free energy expansion describes the strength of field disorder and governs the first-order character of the transition.^37,38,40^ Consequently, the disorder imparts heterogeneous internal stresses within the network that theoretically push the N–I transition toward supercriticality.^38^ Experimental studies show that for a supercritical transition in LCEs, the order parameter undergoes a continuous evolution and the isotropic state is not completely achieved, resulting in a nematic-to-paranematic transition.^41,42^ In addition, bond disorder stems from network heterogeneities, including network strand length dispersity, inconsistent junction functionality, and loop defects, leading to distributed local transition temperatures that convolute to manifest as a broadened phase transition across a bulk sample.^37,39^

Recently, the emergence of a large array of click chemistries^43–46^ has provided straightforward access to LCEs with reduced structural heterogeneities. One such LCE fabrication method involves an end-linked cross-linking scheme that decouples the polymerization of network strands from the cross-linking reaction by incorporating multifunctional cross-linker molecules for network junction sites. The cross-linker molecule enables the synthesis of LCEs with improved control over junction functionality relative to networks cross-linked through acrylate end groups.^12,21,23,25,31,32^ By this method, a thiol-Michael addition reaction is employed to synthesize thiol-terminated LC oligomers, which are subsequently cross-linked with allyl-functionalized junction molecules.

While rarely explicitly addressed, networks cross-linked through reaction of oligomers with acrylate end groups exhibit poorly defined N–I transitions relative to networks cross-linked through the reaction of oligomer end groups with tri- or tetrafunctional junction molecules.^23,47^ Theoretical examinations of the noncross-linked vs cross-linked states of these materials often do not consider the impact of the cross-linker species, which can occupy significant volume fractions of the network at high concentrations, on nematic order.^31,48,49^ Further, network defects and differences in network topology that are influenced by the choice of cross-linker and conditions of the cross-linking reaction are rarely taken into consideration.^50,51^ The simultaneous control of junction functionality and introduction of nonmesogenic cross-linker species in this end-linked cross-linking scheme highlights how nuanced coupling of compositional and topological effects can play a critical role in determining LCE phase behavior.

Here, we systematically examine the interplay between the network structure and composition on the phase transitions of LCEs, with emphasis on the N–I transition. We leverage a system of end-functionalized main-chain LC oligomers cross-linked with two types of junction molecules of different functionalities (*f*), *f* = 3 and *f* = 4. Specifically, we vary the amount of cross-linker species in the LCEs while the molecular weight of the precursor oligomers is held constant. Through calorimetric and rheological characterization, we articulate an interplay between mesogen–backbone coupling and mesogenic dilution when determining *T*_NI_. At low cross-linker concentrations, we find that the degree of cross-linking and the magnitude of change in *T*_NI_ from precursor oligomers to LCE (Δ*T*_NI_) are linearly correlated. Deviations from this relationship at higher cross-linker loadings suggest that at high concentrations, nonmesogenic diluents influence *T*_NI_ differently between the cross-linked and noncross-linked states. Further, in LCEs with stoichiometric compositions where cross-linker functional groups and oligomer end groups are equimolar, we find that increasing the molecular weight of the network strands drives an increase in *T*_NI_, as expected,^52^ but does not have a substantial impact on the ideal latent heat of the N–I transition per mesogen mass (Δ*H*_NI,mes_) for the LCE system under study.

## Experimental Section

### Materials

The mesogenic monomer 1,4-bis-[4-(6-acryloyloxyhexyloxy)­benzoyloxy]-2-methylbenzene (C6M, 97%) was purchased from Synthon Chemicals GmbH & Co. 2,2′-(Ethylenedioxy)­diethanethiol (EDDT, 97%) and 2-benzyl-2-(dimethylamino)-1-(4-morpholin-4-ylphenyl)­butan-1-one (IG-369, >98%) were purchased from Fisher Scientific, while the cross-linkers 1,3,5-triallyl-1,3,5-triazine-2,4,6­(1*H*,3*H*,5*H*)-trione (TATATO, 98%) and glyoxal bis­(diallyl acetal) (GDA, 95%) were supplied by Sigma-Aldrich. 2,6-Di-*tert*-butyl-4-methylphenol (BHT, 99.8%) and triethylamine (TEA, 99.9%) were purchased from Acros Organics. Dichloromethane (DCM, >99.5%) was purchased from Fisher Scientific, and toluene (>99.5%) was purchased from Sigma-Aldrich. All chemicals were used as received without further purification.

### Synthesis of LCEs

Mesogenic monomer C6M (2 g), dithiol chain extender EDDT, and radical inhibitor BHT (2 wt %, 20 mg) were added, in order, into a 20 mL amber glass vial (DWK Life Sciences Wheaton). BHT was used to inhibit premature thermal and UV-induced cross-linking. Subsequently, the cross-linker, either trifunctional TATATO or tetrafunctional GDA, was added volumetrically through concentrated solutions assuming ideal mixing (0.988 g of TATATO dissolved in 1 mL of DCM, 0.833 g of GDA dissolved in 1 mL of DCM). Lastly, the photoinitiator IG-369 (1.5 wt %) and a thiol-Michael addition catalyst TEA (1 wt %) were added, with the combined mass of C6M and EDDT used as the basis. The amounts of EDDT and TATATO/GDA added depend on the desired molecular weight of the LCE precursor oligomer and cross-linking stoichiometry. For example, to prepare a sample with a target degree of polymerization of 13 units (C6M + EDDT) and a stoichiometric ratio of trifunctional cross-linker, 2 g of C6M, 632.3 mg (565 μL) of EDDT, and 82.3 mg of TATATO (83.32 μL of concentrated solution) were used. A detailed list of the amounts used for each LCE composition is provided in Table S1.

The reaction mixture was then placed in a bath filled with aluminum beads set to 90 °C. The mixture was heated for 9 min until melted before being vortexed for 1 min. The reaction vials were transferred to a silicone oil bath set to 65 °C for the thiol-Michael addition reaction to proceed for 24 h, yielding an off-white melt of the thiol-terminated alternating co-oligomer of C6M and EDDT, which serves as a precursor oligomer to the LCE. TEA was then evaporated in the 20 mL amber vial in vacuo from the precursor oligomer using a vacuum oven set to 80 °C and 30 inHg. For a typical batch size with 2 g of C6M, a 2 h evaporation time was used. While small quantities of the cross-linker may be removed due to vacuum, this was considered negligible due to the viscosity of the polymer melt and relatively high boiling points of TATATO and GDA (311 and 297 °C at atmospheric pressure, respectively). The mixture containing the LC oligomers, cross-linker molecule, BHT, and photoinitiator is hereafter referred to as the “precursor oligomer”.

The precursor oligomer was cross-linked on a rheometer (Anton Paar MCR 501) equipped with a quartz Peltier plate accessory (P-PTD 200/GL) coupled to a UV source (OmniCure S2000 UV) equipped with a 365 nm filter (Omnicure, 019-01045R). The precursor oligomer (∼300 mg) was transferred onto the rheometer and heated to 90 °C to ensure cross-linking in the isotropic phase. Using a parallel-plate geometry (20 mm diameter), the gap size was set to 500 μm, after which the oligomer melt was allowed to relax (5–15 min depending on the molecular weight) until the normal force measured by the rheometer was ∼0 N. Excess polymer melt was removed from the edges with a spatula. The melt was then irradiated with UV light for 8 min at an irradiance of 36 mW cm^–2^ to yield the cross-linked LCE.

### Rheological Measurements

During the LCE preparation process, the kinetics of the thiol–ene cross-linking reaction was monitored by continuous measurement of the rheological properties with time (rheological instrument described in the previous section). Storage and loss moduli were measured through oscillatory rheology at a frequency of 1 Hz and an amplitude of 0.5% in the isotropic state (90 °C). Prior to UV exposure, the storage and loss moduli were monitored for 2 min to establish the baseline properties of the precursor oligomer. The moduli were reported as an average of three replicates for each composition, and the errors were estimated by the standard deviation between the three runs. Average *G*′ values and their respective standard deviations are given in Tables S2, S3, and S4 for each composition examined.

### Differential Scanning Calorimetry

The thermal phase transitions of both the precursor oligomers and LCEs were assessed by differential scanning calorimetry (DSC, TA Instruments Discovery DSC 2500). The oligomer precursor or LCE (∼5 mg) was loaded into aluminum pans (TA Instruments Tzero Pans) that were sealed and pinholed to allow the evaporation of any volatile liquids during heat cycles. The samples were subjected to three heating/cooling cycles (heating to 120 °C followed by cooling to −75 °C) at a rate of 10 °C min^–1^. The reported thermal transitions were taken from the final (third) heat cycle. Like the rheological measurements, for each composition, three independent samples were prepared, and the transitions and associated error were taken as the average and standard deviations, respectively, of these three measurements. Average *T*_NI_ and Δ*H*_NI_ values with their respective standard deviations are given in Table S2, Table S3, and Table S4 for each composition examined.

### Wide-Angle X-ray Scattering

Wide-angle X-ray scattering (WAXS) was performed using a Xenocs Zeuss 3.0 benchtop X-ray beamline system equipped with a Cu Kα X-ray source and a DECTRIS EIGER2 R 1M pixel 2D detector system. All measurements were taken in the “high-resolution” configuration, corresponding to a beam size of 0.3 mm by 0.3 mm. The sample-to-detector distance was calibrated using lanthanum hexaboride (LaB_6_).

For WAXS measurements of the precursor oligomers, the samples were enclosed between two layers of 8 μm-thick Kapton film within a brass washer (M5, 0.65 mm thickness). The washer was first glued onto a Kapton piece with heat-resistant epoxy and allowed to cure. Once the epoxy had set, the oligomer was deposited into the brass washer on a hot plate set to 80 °C. The washer was then sealed with an additional piece of Kapton. The Kapton-enclosed oligomer was then placed in a gasket compatible with a Linkam HFSX heating/cooling stage. The oligomers were heated to 75 °C at a rate of 25 C/min above their respective *T*_NI_s and cooled at 5 C/min to temperatures corresponding to their expected nematic and smectic phases. The temperatures were selected with reference to DSC measurements. At each temperature, the sample was allowed to equilibrate for 60 s before being exposed to X-rays for 5 min.

LCE disks, taken from in situ rheology, were cut into rectangular strips (20 mm × 7 mm × 0.5 mm) and mounted to a Linkam MFS tensile stage equipped with a 200 N load cell. WAXS measurements for the strips were taken in both the stretched and unstretched states to determine the mesogen order. For the unstretched measurements, each sample was heated at 25 °C/min to the isotropic state at 100 °C and subsequently cooled at a rate of 5 °C/min to temperatures corresponding to the expected nematic and smectic phases from DSC. At each temperature, the sample was exposed to X-rays for 5 min. The sample was then heated at 25 °C/min to 80 °C (once again above the *T*_NI_) to reset any thermal history and then cooled at 5 °C/min to the nematic phase. Each sample was then stretched to 150% engineering strain to induce chain alignment. The sample was further cooled at 5 °C/min to the expected smectic phase while being held under 150% engineering strain. The sample temperature was allowed to equilibrate for 60 s after each heating/cooling step. At each temperature, the strained LCEs were exposed to X-ray irradiation for 5 min.

Because the Linkam MFS tensile stage was not equipped with calibrants, an additional LCE sample was mounted on the calibrated Linkam HFSX stage to acquire a reference scattering pattern in the determination of peak positions for the LCEs. All LCE peak positions are reported as corrected values.

### Polarized Optical Microscopy

Polarized optical microscopy (POM) measurements were conducted using a Zeiss Axioscope A1 upright optical microscope in transmission mode under a bright-field configuration equipped with an LD Epiplan Neofluar 50×/0.55 DIC objective. POM samples were prepared by sandwiching a drop of oligomer melt between glass coverslips while being heated on a hot plate set to 90 °C. To observe thermal transitions in situ, the oligomer sandwiches were subject to heating/cooling runs on a Linkam FTIRSP600 heating stage equipped with a Linkam T95PE sample stage. Active cooling was supplied to the stage via a Linkam LNP95 liquid nitrogen pump. Samples were heated above their *T*_NI_ and slowly cooled at 0.1 °C/min to allow development of birefringent textures under crossed polarizers.

### Gel Fraction Measurements

Gel fractions of the LCEs were taken by recording the initial mass of the networks before swelling them in toluene for 4 days. On each day, the LCEs were transferred to fresh toluene. The swollen LCEs were then dried in a vacuum oven at 90 °C and 30 inHg for 24 h, and the final mass was recorded.

### Precursor Oligomer Molecular Weight

The molecular weight distributions of the precursor oligomers were characterized by gel permeation chromatography (GPC, Water Alliance HPLC equipped with an E2695 separation module). The oligomer solutions (3–5 mg mL^–1^, injection volume of 40 μL) were separated through a Tosh TSKgel SuperHZM-N column (molecular weight range of 200–700,000 Da) using chloroform with 0.25% TEA as a mobile phase at a flow rate of 0.35 mL min^–1^. The eluted oligomers were analyzed using both refractive index (Waters 24104 differential refractometer) and UV detectors (Waters 2998 photodiode array detector) relative to polystyrene standards.

## Results and Discussion

### Controlling LCE Composition and Structure through Cross-Linker Concentration and Network Strand Molecular Weight

The N–I transition in LCEs is known to be impacted by several factors: cross-link density,^53,54^ order at network formation (orientational genesis),^32,53^ and nonmesogenic diluents.^31^ The deconvolution of these effects of cross-linking is critical to understanding the impact of the network structure on LCE thermal and mechanical properties. To this end, we investigated two series of LCEs. In the first series (series 1), the length of the precursor oligomer, which controls cross-link density through the distance between cross-linking junctions (network strand length), was fixed while the concentration of the cross-linker molecule was varied. In the second series (series 2), we tuned the length of the precursor oligomer and maintained an equimolar ratio between oligomer end groups and cross-linker functional groups. By these series, the composition of the LCEs was varied in two distinct ways: the amount of cross-linker molecules added to the LCE and the mesogen fraction of the oligomer precursor chains. As a result of the different methods of changing composition, the network structure is varied by either the degree of cross-linking (series 1) or the length of interjunction network strands (series 2).

By fixing the precursor oligomer length and varying the extent of cross-linking through cross-linker concentration, series 1 probes the combined impact of the network structure and mesogenic dilution on the temperature of the smectic-to-nematic and nematic-to-isotropic transitions. As shown schematically in Figure 1a, at low cross-linker concentrations, the network is expected to be incompletely cross-linked, resulting in dangling chain ends. The degree of cross-linking should increase with cross-linker concentration until a maximum is reached. Beyond this maximum, the cross-link density decreases as the number of cross-linker functional groups is in excess and bridging chains undergo chain extension due to junctions that are not fully substituted. When only two sites of a junction react, the cross-linker molecule no longer contributes to network formation and behaves as a chain extender, thereby decreasing the cross-link density at high cross-linker concentrations. In this series, the cross-linker molecule is also expected to behave as an nonmesogenic defect that dilutes the fractional mesogen content of LCEs to depress *T*_NI_.^31^ The combined effects of the degree of cross-linking and mesogenic dilution dictate the thermal behavior of the LCEs.

**Figure 1:**
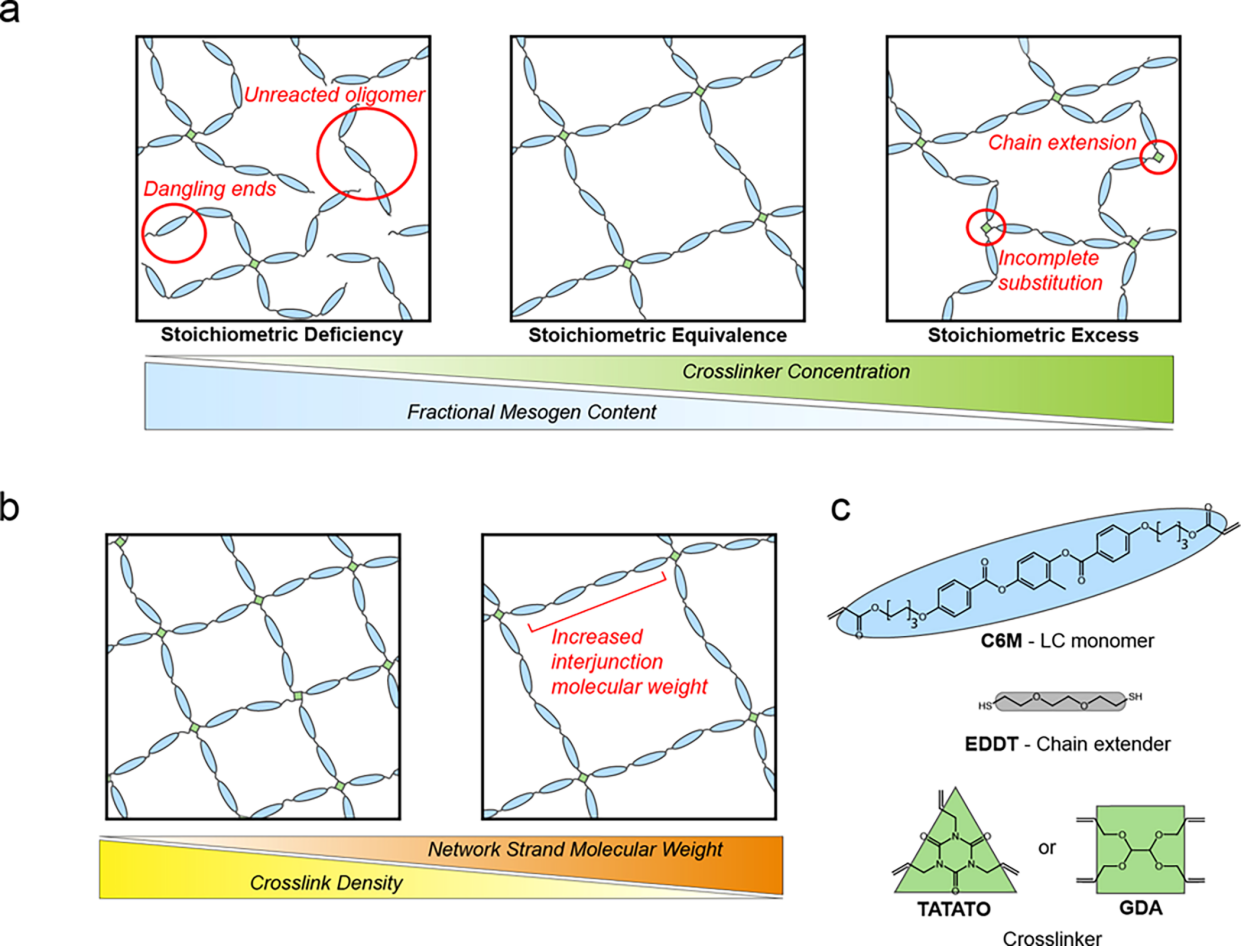
Schematic representations of methods used to control the LCE topology. (a) Series 1: schematic depicting the structural changes in LCEs when the cross-linker concentrations are varied for a given precursor oligomer length. Incomplete cross-linking is observed at low cross-linker loadings, while chain extension and incomplete substitution occur with an excess of cross-linker functional ends. The maximum degree of cross-linking achievable for a given network strand length is reached at an intermediate cross-linker concentration. As more isotropic cross-linker is introduced, the fractional mesogen content of the LCEs decreases. (b) Series 2: schematic of LCEs with decreasing cross-link density induced by increasing the interjunction molecular weight via network strand molecular weight and maintaining a stoichiometric equivalence of functional ends. (c) Chemical structures of the primary components of the LCEs investigated in this study. The mesogenic monomer (C6M, blue oval) is first reacted with the chain extender (EDDT, gray rectangle) through step growth polymerization to yield precursor oligomers, which are then photo-cross-linked by a thiol–ene mechanism with a tri- or tetrafunctional cross-linker (TATATO and GDA, respectively, green triangles and squares). Schematics are depicted as networks synthesized with tetrafunctional cross-linkers.

Series 2 examines the effect of tuning network strand length while maintaining a stoichiometric ratio between the precursor oligomer chain ends and cross-linker functional groups (illustrated in Figure 1b). Variation of the average molecular weight between cross-linking junctions and therefore, cross-link density, has been shown to play an important role in determining *T*_NI_ through network strand mesogen fraction and a chain length-dependent mismatch of entropic penalties imposed on the nematic and isotropic phases.^55^ Further, we examine the effect of network strand length on the breadth and integrated enthalpy of the nematic-to-isotropic transition to assess the first-order character of the N–I transition.

LCEs with independently controlled network strand lengths and degrees of cross-linking were fabricated using a two-step method.^12,23,56^ In the first step, a dithiol chain extender (EDDT) was co-oligomerized with a diacrylate liquid crystalline monomer (C6M) through a thiol-Michael addition reaction to synthesize thiol-capped LC oligomers. The length of the oligomer chain was controlled by tuning the ratio between EDDT and C6M. Through this ratio, the number-average molecular weight (*M*_n_), represented here by the theoretical number of mesogens per chain (*n*), can be predicted through Carothers’ equation for nonstoichiometric step growth polymerization assuming complete conversion.^57,58^ Additionally, the mesogen mass fraction of each oligomer chain varied with the total length. For a chain with *n* mesogenic units, there are *n* + 1 corresponding spacer units. The molar mesogen fraction *n*/(2*n* + 1) therefore increases with the number of mesogens between network junctions.

The oligomer’s thiol chain ends were photo-cross-linked through a thiol–ene reaction with either a trifunctional (TATATO) or a tetrafunctional (GDA) cross-linker. The degree of cross-linking was tuned by varying the ratio between the total number of allyl groups from the cross-linker and the predicted number of thiol end groups of the LC precursor oligomer. The chemical structures for C6M, EDDT, and the cross-linkers TATATO and GDA are shown in Figure 1c. Triethylamine, which was used as a thiol-Michael addition catalyst, was evaporated from the oligomer melt prior to cross-linking to eliminate its possible contribution as a nonmesogenic small-molecule additive.

Because LCE thermal properties are strongly dependent on the order parameter at network formation,^1,53,59,60^ the thiol–ene cross-linking reaction was initiated by irradiating with UV light (365 nm) while the oligomer melt was in the isotropic state (90 °C), stabilizing the random polymer backbone conformations through network formation. In this approach, known as “isotropic genesis”, the order parameter is expected to be nearly zero during cross-linking and contributions from orientational order at cross-linking are minimized. The absence of an external alignment field resulted in opaque LCEs at room temperature indicative of a polydomain texture due to light scattering from misaligned birefringent micrometer-scale domains of local nematic ordering.^1,61–63^ By contrast, nematic genesis LCEs are cross-linked below *T*_NI_, stabilizing the nematic order present at network genesis. The heightened residual anisotropy manifests as a loss in “softness”, resulting in a polydomain–monodomain transition over a wide range of stress values as well as higher *T*_NI_s than isotropic genesis LCEs.^1,64^

### Competition between Cross-Link Density and Orientational Genesis Determines *T*_NI_ for a Given Composition

Probing the interplay of network structure and thermal properties requires quantifying mechanical changes due to cross-linking. To achieve this, the kinetics of the thiol–ene reaction were monitored by measuring the shear storage (*G*′) and loss (*G*″) moduli during UV irradiation. In Figure 2a, *G*′ and *G*″ are plotted as a function of time for a precursor oligomer (*n* = 6) with no cross-linker (orange lines) and a stoichiometric amount of GDA (green lines). When no cross-linker is added, thiol–ene cross-linking is not possible, but *G*′ increases slightly during UV irradiation. This is likely due to bimolecular recombination of thiyl radicals in the oligomer melt, effectively causing chain extension.^65^ In contrast, when a stoichiometric amount of GDA is present, the thiol–ene reaction causes a rapid increase in *G*′ upon exposure to UV with the crossover point between *G*′ and *G*″ reached in less than 5 s (inset of Figure 2a). *G*′ reaches 94% of its maximum value and plateaus by ∼5 min of UV irradiation, reaching a final value of 1.81 × 10^5^ Pa after 8 min. The final *G*′ is 2 orders of magnitude larger than the initial storage modulus (1.24 × 10^3^ Pa). Although the reactive moieties are likely not completely consumed in this limit, the limited mobility of the cross-linking junctions and chain ends inhibits further cross-linking.

**Figure 2:**
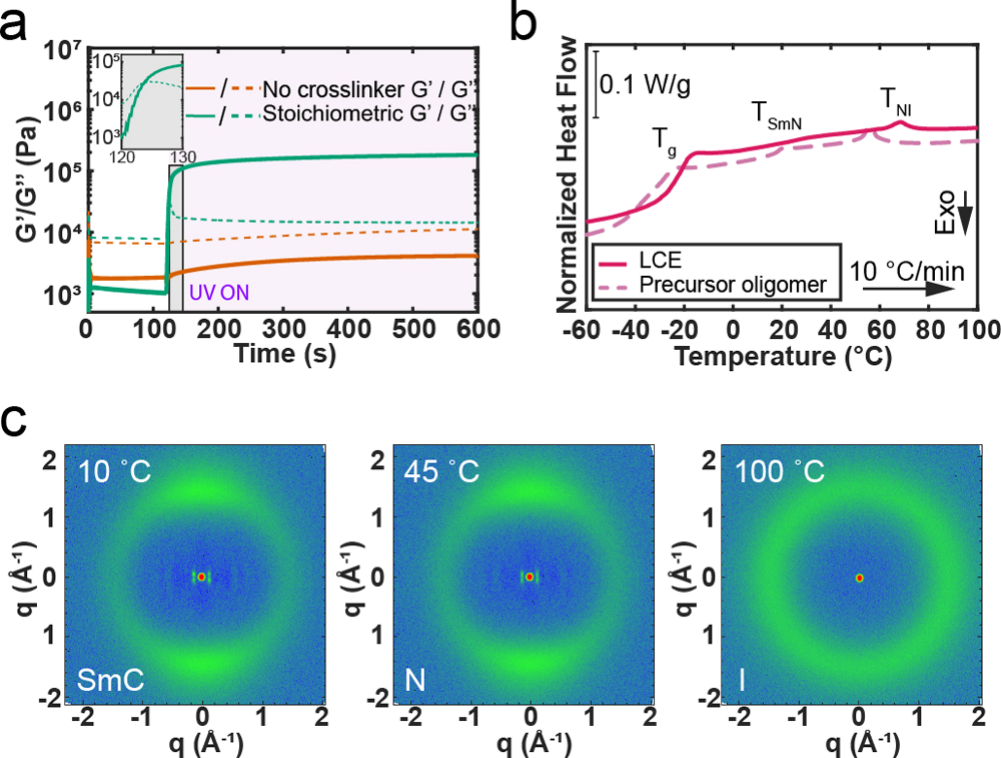
Representative change in rheological and thermal properties during cross-linking of a precursor oligomer with tetrafunctional GDA. (a) Change in storage (*G*′, solid lines) and loss moduli (*G*″, dashed lines) prior to and during UV irradiation for a precursor oligomer with no GDA (orange lines) and a stoichiometric amount of GDA (green lines). Inset: crossover point of *G*′ and *G″* for stoichiometric GDA LCE. The purple shaded area represents the time at which the sample is irradiated with UV light. (b) Third DSC heating scan of the precursor oligomer with stoichiometric amounts of GDA prior to (dashed line) and after (solid line) UV irradiation, showing the broadening of the Sm–N and N–I transitions after cross-linking. (c) 2D WAXS patterns of the stoichiometric GDA LCE in the smectic C (SmC), nematic (N), and isotropic (I) phases. 2D patterns in the smectic C and nematic phases shown were taken after stretching the LCE to 150% engineering strain.

In addition to changes in rheological behavior due to the cross-linking reaction, the change in phase behavior that accompanies the transition from discrete LC oligomers to a cross-linked LCE network can be characterized through DSC. Figure 2b shows representative DSC traces during the third heating scan (10 °C min^–1^) of a stoichiometric GDA composition before and after cross-linking. This sample is equivalent to the stoichiometric composition shown in the rheological characterization in Figure 2a. The traces for both the precursor oligomer and the LCE show three distinct transitions: glass transition, smectic-to-nematic transition (Sm–N), and N–I transition (Figure 2b). These transitions are reversible upon cooling, as shown in Figure S1. Upon cross-linking, broadening of the Sm–N and N–I transition is observed through an increase in the endothermic peak’s full width at half-max (fwhm). For Sm–N, the fwhm increases from 6.1 °C to undefinably broad and from 5.4 to 8.4 °C for N–I. This broadening is attributed to the internal stresses imposed on the network through the network junctions and local variation in transition temperatures due to network heterogeneities.

To verify the phase sequence and the identity of the expected smectic phase, 2D WAXS patterns of the same stoichiometric GDA LCE at temperatures corresponding to each expected phase were collected and are shown in Figure 2c. Above *T*_NI_ (100 °C), the isotropic phase is evident through an amorphous halo at 1.45 Å^–1^ and minimal higher-order reflections. Below *T*_NI_ and above *T*_SmN_ at 45 °C, the LCE was stretched to a 150% engineering strain to induce a monodomain. The corresponding 2D pattern displayed two bright spots at 1.45 Å^–1^ perpendicular to the stretching direction, indicative of aligned nematic domains. When the mixture was cooled below *T*_SmN_ at 10 °C, four bright spots on diagonal axes to the stretching direction at 0.20 Å^–1^ were observed while retaining the two bright spots perpendicular to the stretching direction at 1.50 Å^–1^. The four inner spots were attributed to the length scale of smectic C (SmC) layers arranged in chevrons throughout the sample,^33,66^ thus confirming the phase sequence of isotropic–nematic–smectic C. Similar scattering patterns were observed in the respective precursor oligomer in Figure S2a,b, characterized by an inner halo (0.14 Å^–1^) indicative of the development of a smectic phase when cooled from the nematic phase. Scattering patterns for LCEs with varied amounts of either TATATO or GDA are given in Figures S3 and S4, respectively, to demonstrate the universality of the phase behavior in the *n* = 6 system. While POM was also attempted to confirm phase behavior, the slow texture coarsening of the smectic phase due to the viscosity of the oligomers^67–69^ rendered it indistinguishable from the nematic phase despite a slow cooling rate of 0.1 °C/min (Figure S2c).

For all LCE samples in this study, *T*_NI_ increases upon cross-linking. For the composition shown in Figure 2b, *T*_NI_ increases from 55.6 to 68.5 °C. This increase is contrary to the prediction of a previous theoretical treatment of the N–I transition for an LCE cross-linked in the isotropic state.^53^ To account for the genesis of the LCE (isotropic or nematic), this theory incorporates an additional elastic free energy term to the LdG free energy expansion that depends on the scalar nematic order parameter (*S*) during network formation. *S*, defined in eq 1, represents the ensemble-averaged angle between the long axis of each mesogen and the global nematic director.

$$S=\left\langle \frac{3}{2}\cos^{2}\theta -\frac{1}{2}\right\rangle$$ 1

Minimization of the LdG free energy expansion including the additional elastic free energy term results in a Δ*T*_NI_ dependent on the order parameter at network formation (*S*_0_) and at the N–I transition (*S*_NI_), as shown in eq 2.

$$\begin{array}{c} \Delta T_{\mathrm{NI}}=\frac{3\mu \alpha^{4}}{a_{0}}\left(S_{0}^{2}-\frac{1}{2}S_{\mathrm{NI}}^{2}\right) \end{array}$$ 2

Here, μ is the elastic modulus, α is a dimensionless proportionality constant that accounts for differences in the orientational order of the mesogens relative to the order of the polymeric backbone, and *a*_0_ is a LdG free energy constant. From eq 2, this LdG treatment suggests that the *T*_NI_ should decrease on cross-linking (Δ*T*_NI_ < 0) for an isotropic genesis LCE (*S*_0_ = 0). This prediction is inconsistent with the increase in the *T*_NI_ observed in the current study.

An alternative treatment to account for mechanical field effects in cross-linked LCEs introduces additional free energy terms to the LdG expansion that represent external stresses, elastic strain energy, and the coupling of strain of the network and mesogen order.^54,70^ In the absence of external fields, a spontaneous strain that is coupled to the nematic order is required to minimize the LdG free energy. Minimization of the free energy expansion over *S* in the absence of an external stress field leads to eq 3:

$$\Delta T_{\mathrm{NI}}=\frac{U^{2}}{a_{o}\mu }$$ 3

Here, *U* represents the coupling between the mesogens and the polymer backbone and is proportional to μ. Following eq 3, *T*_NI_ always increases for an LCE compared to its precursor oligomer regardless of genesis due to the elastic coupling between the network chain conformation and mesogen orientation. *U* is proportional to cross-link density, indicating that a larger change in *T*_NI_ from oligomer to LCE is expected for more tightly cross-linked networks.

These two theoretical treatments appear to yield contradicting trends, and experimental results have been likewise inconsistent with regard to the effects of isotropic genesis. Some studies have found that LCEs cross-linked in the nematic and isotropic states exhibit an increase and decrease in *T*_NI_, respectively,^2,71^ while other studies, including this work, have shown an increase in *T*_NI_ despite an isotropic genesis.^52,72^ Broadly, the former studies are of side-chain LCEs while the latter is of main-chain LCEs.

The apparent difference between these results can be explained by the degree of coupling between the mesogens and the polymer backbone. In main-chain LCEs, which have a particularly high degree of coupling between mesogens and the backbone,^73^ the coupling effects can supersede the depressive effects of isotropic genesis, leading to a net increase in *T*_NI_. To confirm the combined effects of orientational genesis and mesogen–backbone coupling, we measured *T*_NI_ of an LCE prepared with the same composition as that shown in Figure 2b but cross-linked in the nematic state at room temperature. In this case, both theories predict an increase in *T*_NI_, leading to a higher *T*_NI_ than that of isotropic genesis. DSC scans for LCEs with an isotropic and nematic genesis is shown in Figure S5a,b. These scans demonstrate consistently higher *T*_NI_s for the nematic genesis LCEs compared to the isotropic genesis LCEs irrespective of cross-linker functionality (trifunctional TATATO: 69.1 °C vs 62.1 °C, tetrafunctional GDA: 74.9 °C vs 65.0 °C). Therefore, while eq 2 and eq 3 are not directly comparable, we can infer that a competition arises between effects due to the order at cross-linking and those due to mesogen–backbone coupling.

### Mesogen–Backbone Coupling and Mesogenic Dilution Compete to Determine *T*_NI_

To probe the role of the cross-linker as a nonmesogenic diluent in combination with mesogen–backbone coupling due to network formation, isotropic genesis LCEs were synthesized with cross-linker concentrations below, above, and corresponding to stoichiometric equivalency. While not all compositions result in a fully cross-linked material, all post-UV-treated materials are referenced as LCEs. The length of the precursor oligomers was fixed for all samples at *n* = 6, and three replicates were examined for each composition.

The evolution of *T*_NI_ as a function of cross-linker concentration for the precursor oligomers and the LCEs is shown for TATATO and GDA compositions, in Figure 3a,b. DSC analysis of the precursor oligomers reveals that dilution of mesogen content results in a suppression of *T*_NI_ with increased cross-linker concentration for both TATATO and GDA. For the precursor oligomers, the cross-linker acts only as a nonmesogenic additive, resulting in the observed *T*_NI_ depression. Surprisingly, the decrease in *T*_NI_ with concentration appears to plateau beyond 0.12 mol equiv (TATATO) and 0.083 mol equiv (GDA), a distinction that becomes important when we later compare these to the cross-linked materials of equivalent composition.

**Figure 3:**
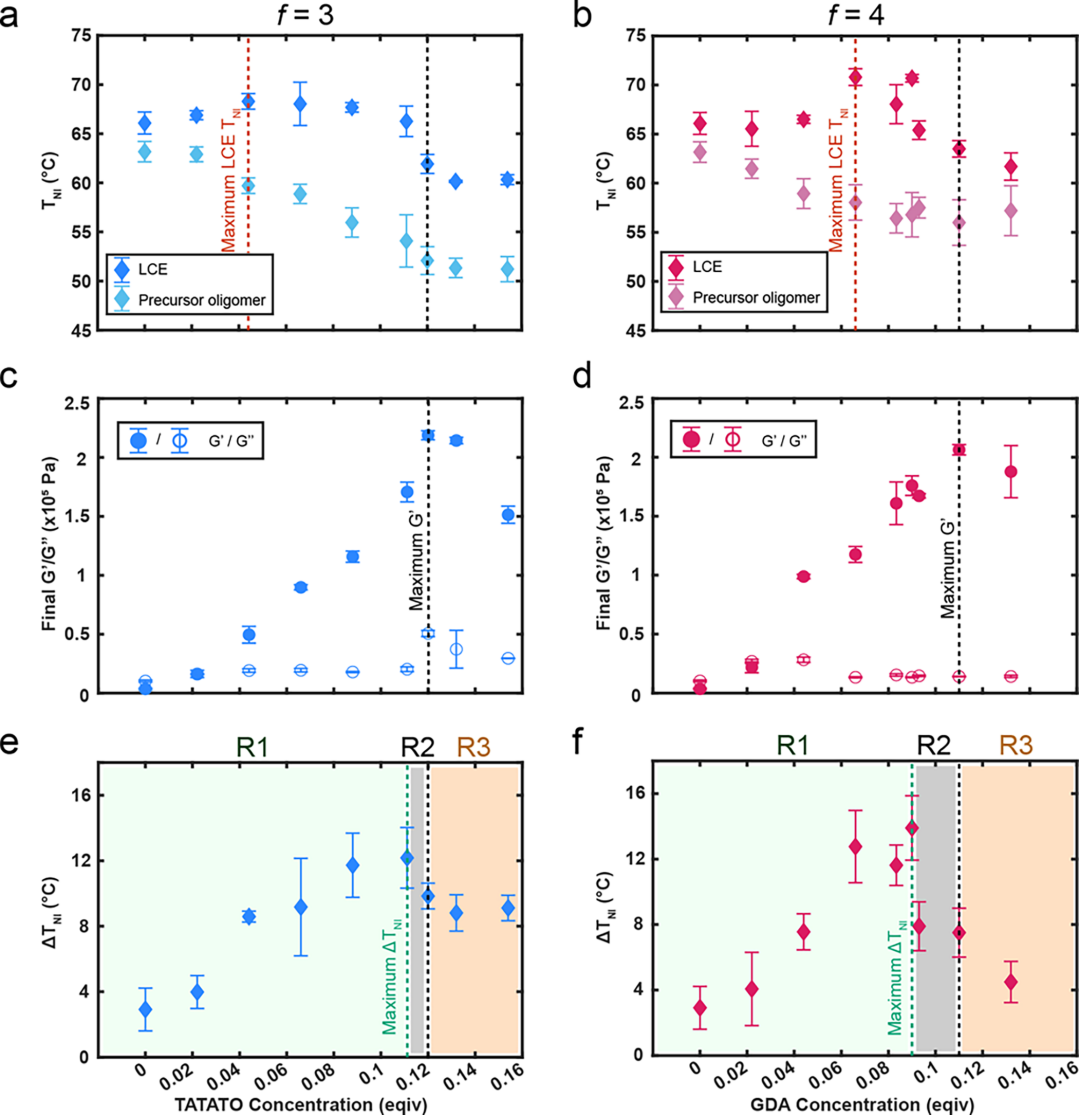
*T*_NI_ and final storage and loss moduli as a function of cross-linker concentration show combined effects of nonmesogenic diluents and mesogen–backbone coupling. Δ*T*_NI_ reduces contributions from mesogenic dilution. Cross-linker functionality is denoted as *f*. TATATO (*f* = 3) and GDA (*f* = 4) are added in molar equivalents with respect to C6M. (a) *T*_NI_ of TATATO precursor oligomers and LCEs. (b) *T*_NI_ of GDA oligomers and LCEs. (c) Final *G*′ and *G*″ for TATATO LCEs and (d) GDA LCEs. (e) Δ*T*_NI_ of TATATO LCEs and (f) GDA LCEs. For all plots, the cross-linker concentrations corresponding to the maximum *T*_NI_, *G*′, and Δ*T*_NI_ are denoted as orange, black, and green dashed lines, respectively. Three regimes corresponding to differing relations between trends in Δ*T*_NI_ and *G*′ are shaded green, gray, and orange and are, respectively, labeled as R1, R2, and R3.

For the LCEs, *T*_NI_ is a more complex function of cross-linker content compared to the precursor oligomers due to the additional contribution of mesogen–backbone coupling from cross-linking. As the degree of cross-linking is increased, the degree to which the network polymer backbone conformation is associated with the orientational order of the mesogens is augmented, thus resulting in an upward shift in *T*_NI_ due to elastic contributions.^54^ For both TATATO and GDA LCEs, *T*_NI_ initially increases with cross-linker concentration up to a maximum, beyond which it decreases with concentration. Similar trends are observed for the Sm–N transition temperature (Figure S6a,c). The competition between mesogenic diluent effects and mesogen–backbone coupling can be visualized in Figure 4. Isotropic additives occupy volume within the system to dilute mesogenic content and disrupt nematic order, thereby having a depressive impact on *T*_NI_ (Figure 4a).^31^ Oligomerization and cross-linking restrict mesogen mobility through increased backbone–mesogen coupling (Figure 4b),^54,70^ thereby rendering a phase transition more difficult as perturbing mesogen orientation now constitutes also perturbing the polymer chain and cross-linked network. While the increased coupling has an enhancing effect on *T*_NI_, it is in competition with the simultaneous dilution of mesogen fraction by the addition of spacer monomers and cross-linker species.

**Figure 4:**
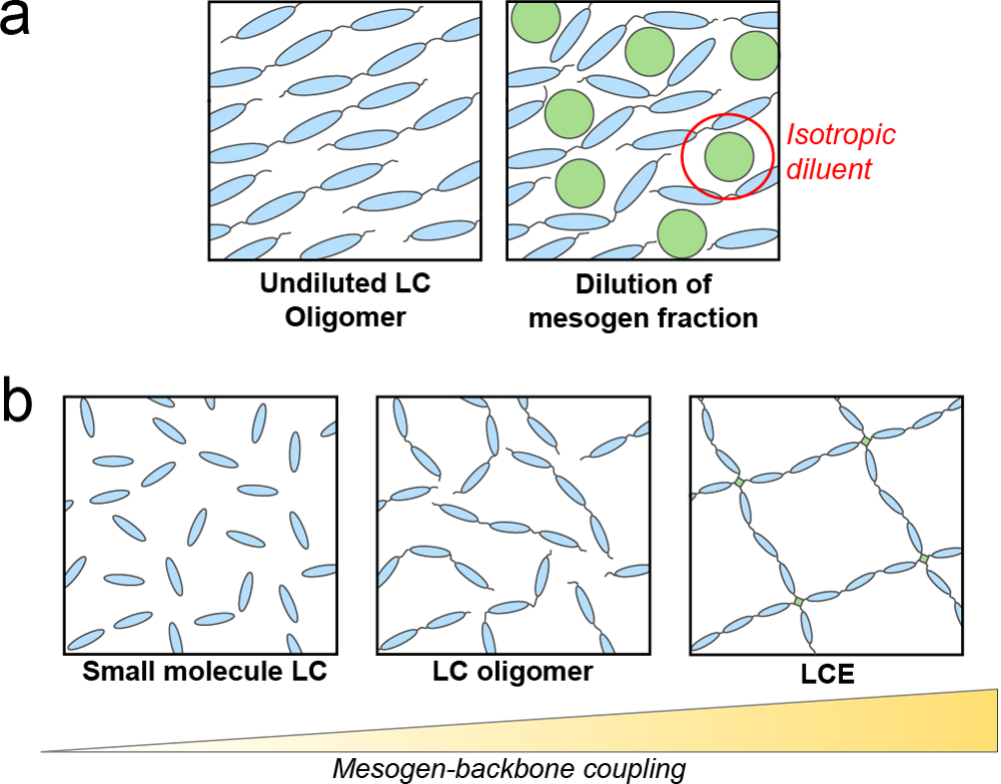
Schematic representation of mesogen fraction dilution effects and mesogen–backbone coupling effects. (a) Isotropic diluents (green circles) can disrupt nematic order. (b) Oligomerization and cross-linking increase the degree of mesogen–backbone coupling relative to small-molecule LCs. Sizes of the isotropic diluent and mesogens are not drawn to scale.

To understand the shifts in *T*_NI_ relative to the evolution of mesogen–backbone coupling, the degree of cross-linking can be monitored through the storage modulus (*G*′, filled circles) of the LCEs (Figure 3c,d). The storage modulus is proportional to the concentration of elastically effective strands.^51,74,75^ The final modulus was taken as the last data point of the in situ UV rheology of the thiol–ene cross-linking reaction. The initial upward trend in *G*′ from 0 to 0.12 equiv and from 0 to 0.11 equiv for TATATO and GDA, respectively, is due to an increased degree of cross-linking as chains are incorporated into cross-link junctions. We remark that the maximum *G*′ (*G*′_max_) composition, corresponding to the most cross-linked state, appears beyond 0.11 and 0.083 equiv of the cross-linker, the ratio at which TATATO- and GDA-containing networks are expected to achieve stoichiometric compositions. Full functionalization of network junctions is unlikely due to steric and diffusive constraints, and an excess of cross-linker molecules is required to reach the maximum *G*′. At cross-linker concentrations beyond *G*′_max_, a decrease in *G*′ is observed. Here, the excess of cross-linker functional groups introduces chain extension and lowers the cross-link density. Notably, gel fractions of TATATO-synthesized LCEs are significantly lower (maximum of 0.79) than those of GDA-synthesized networks (maximum of 0.92) even in the “most cross-linked” states (Figure S7a,b). Despite this difference, TATATO and GDA networks exhibit similar *G*′_max_ values, reflecting that despite clear differences in network structure and gel fraction possibly attributed to a combination of -ene reactivity and cross-linker functionality,^76^ the average density of elastically effective strands is similar. All DSC and rheology measurements are performed on samples retaining the solvent-extractable fraction. Unlike the storage moduli, the loss moduli (*G*″) stay relatively constant irrespective of cross-linker loading. Additionally, the *T*_NI_ peak fwhm of the LCEs increases with cross-linker concentration (Figure S8a,b), further supporting the observed evolution of the degree of cross-linking as quenched disorder is increased with the number of network junctions.^38,41,42,77^ The change in fwhm beyond the cross-linker concentration corresponding to *G*′_max_ is less pronounced and displays a shallow decrease as cross-link density is reduced for the GDA LCEs while the trend for the TATATO LCEs is less conclusive. The glass transition temperature increases and shows a shallow decrease at excess cross-linker concentration in support of the increase in chain extension in this regime (Figure S6b,d).

As another measure of disorder,^41,42,78^ the N–I transition latent heat (Δ*H*_NI_) and Δ*H*_NI_ normalized by mesogen mass fraction (Δ*H*_NI,mes_) are given as a function of cross-linker concentration in Figures S8c,d and 8e,f, respectively. The normalization of Δ*H*_NI_ by the C6M mass content reduces the influence of liquid crystalline composition on enthalpic interactions to quantify the first-order character of the N–I transition. Here, we observe that Δ*H*_NI_ generally decreases with cross-linker concentration for oligomers and LCEs for both TATATO and GDA. For GDA, Δ*H*_NI_ is comparable between oligomer and LCE at low cross-linker concentrations but begins to diverge as more cross-linker is added. Unlike GDA, oligomers with TATATO exhibit a higher Δ*H*_NI_ than TATATO LCEs throughout the investigated cross-linker concentration range, possibly due to different network structures between TATATO and GDA LCEs stemming from cross-linker functionality and -ene reactivity.^76^

To begin relating mesogen–backbone coupling to *T*_NI_, we can initially qualitatively compare the trends of *G*′ and *T*_NI_. At low cross-linker concentrations, there is likely limited network formation as *G*′ begins to increase; regardless, the formation of higher-order (*f* > 2) junctions distributed through the material is sufficient to increase *G*′ and thus increase the degree of mesogen–backbone coupling to increase *T*_NI_. At these cross-linker concentrations, we can consider the impact of the cross-linking reaction on *G*′ as similar to converting a fraction of the linear oligomers to longer oligomers (*f* = 2) or star oligomers (*f* = 3 and 4).^79,80^ As the concentration of cross-linker molecules is further increased, the fractional mesogen content of the LCE decreases. At cross-linker concentrations beyond the composition for *G*′_max_, the depression of *T*_NI_ is predominantly due to the combination of mesogenic dilution and reduced cross-link density due to anincrease in chain extension.

Interestingly, *T*_NI,max_ and *G*′_max_ are at different cross-linker concentrations for both TATATO and GDA, demonstrating that *T*_NI_ is determined by the combined effects of mesogenic dilution and the degree of cross-linking (Figure 3a,d, orange and black dashed lines). Fractional mesogen content decreases inversely with cross-linker concentration; this nonlinear decrease in mesogen content likely initially evolves at a faster rate with cross-linker concentration than mesogen–backbone coupling, which may contribute to causing *T*_NI_ to peak prior to the *G*′_max_. In addition, compared to the tetrafunctional GDA, the trifunctional TATATO requires a higher concentration to match a given number of functional groups. Therefore, the mesogenic dilution from TATATO is more apparent at lower cross-linker concentrations than GDA, which leads to the wider discrepancy between *T*_NI,max_ and *G*′_max_ concentration values. The convolution of mesogenic dilution and mesogen–backbone coupling can be observed more explicitly when LCE *T*_NI_ is shown as a function of *G*′ in Figure S9a. Here, as the cross-linker concentration increases, *T*_NI_ deviates from linearity with *G*′ as *T*_NI_ is lowered by mesogenic dilution despite an increasing degree of cross-linking.

### Storage Modulus and Δ*T*_NI_ Correlate Structural Changes to LCE Thermal Properties

The effects of mesogenic dilution can be reduced by considering Δ*T*_NI_, the difference in *T*_NI_ between a precursor oligomer and the corresponding LCE (Figure 3e,f). Compared to *T*_NI_, the composition corresponding to the maximum Δ*T*_NI_ (Δ*T*_NI,max_) (Figure 3e,f, green dashed lines) moves closer to the *G*′_max_ composition for both TATATO and GDA but still does not show complete agreement. Three regions (R1, R2, and R3) with distinct relationships between Δ*T*_NI_ and *G*′ can be observed. In R1, both Δ*T*_NI_ and *G*′ increase linearly with cross-linker concentration. Here, the agreement between Δ*T*_NI_ and *G*′ implies that Δ*T*_NI_ is primarily determined by the degree of mesogen–backbone coupling, which increases proportionally with the degree of cross-linking and thereby *G*′. In R2, surprisingly, Δ*T*_NI_ decreases with cross-linker concentration despite an increasing *G*′; we discuss this region in more depth in the following paragraph. Finally, in R3, Δ*T*_NI_ decreases simultaneously with *G*′ as the degree of mesogen–backbone coupling is lowered due to a decrease in the average junction functionality.

The nonmonotonic evolution of Δ*T*_NI_ with cross-linker concentration can be more clearly visualized by examining Δ*T*_NI_ directly plotted versus *G*′ (Figure S9b). At low cross-linker concentrations in R1, *G*′ scales linearly with Δ*T*_NI_. However, as cross-linker concentration is further increased into R2, Δ*T*_NI_ exhibits a steep decrease despite increasing *G*′. The diluent effects of the cross-linker manifest differently in the precursor oligomer compared to the cross-linked LCE, meaning that they do not transfer from the precursor oligomers to the LCE in an additive manner. The steeper decrease in LCE *T*_NI_ compared to the oligomers exhibited beyond the LCE *T*_NI,max_ suggests that the effects of mesogenic dilution are more pronounced in the cross-linked state than the noncross-linked state, possibly due to more permanent distortions of mesogen orientation due to covalent cross-linking as opposed to dormant cross-linker molecules occupying volume in the oligomer melt (Figure 3a,b). These nonidealities in the “subtraction” of mesogenic dilution effects when considering Δ*T*_NI_ seem to appear at higher cross-linker concentrations, and thus, greater care must be taken when correlating *G*′ and Δ*T*_NI_ in this regime. As such, the efficacy of utilizing Δ*T*_NI_ relies heavily on the absolute amount of cross-linker added to the system. For example, a similar series derived from LCEs with network strands longer than those shown here might demonstrate a more ideal match between the compositions leading to concurrent Δ*T*_NI_ and *G*′. Similarly, one with shorter network strands might exhibit less ideal behavior, leading to more deviation between maxima in Δ*T*_NI_ and *G*′.

### Variation of Oligomer Molecular Weight Identifies Maximum LCE Latent Heat Per Gram Mesogen

Previous sections have focused on the role of cross-linker concentration in determining LCE thermal properties. Here, we now investigate the impact of the network strand molecular weight and fractional mesogen content on the phase behavior of the LCEs by leveraging the independent control over precursor oligomer synthesis and cross-linking afforded by the synthesis method. The following experiments focus on LCEs chosen for stoichiometric equivalency between oligomer and cross-linker functional ends, for which the length of the precursor oligomer was varied from *n* = 2 up to *n* = 10 by controlling the ratio of EDDT and C6M. GPC measurements confirm the change in oligomer molecular weight with *M*_n_ ranging from 4.8 to 19.3 kDa with respect to polystyrene standards (Figure S10). The relative molecular weight values are higher than the expected *M*_n_, which ranges from 1.9 to 8.7 kDa when calculated by Carothers’ equation. Differences in hydrodynamic radii between the LC oligomer and polystyrene standards are likely to cause this discrepancy.

*T*_NI_ values for the precursor oligomers and the corresponding LCEs for different network strand molecular weights measured with DSC are shown in Figure 5a. For both TATATO and GDA LCEs, the precursor oligomer *T*_NI_ increases with molecular weight and a similar trend is observed for the LCEs at an elevated *T*_NI_. As established in previous studies, *T*_NI_ becomes more weakly dependent on oligomer molecular weight for the precursor oligomer at longer chain lengths;^81,82^ this trend also carries over to the LCEs investigated here. Because of the similarity in the trends for the two cross-linkers and due to the higher gel fractions uniformly observed in GDA networks, we focus here on GDA oligomers and LCEs.

**Figure 5:**
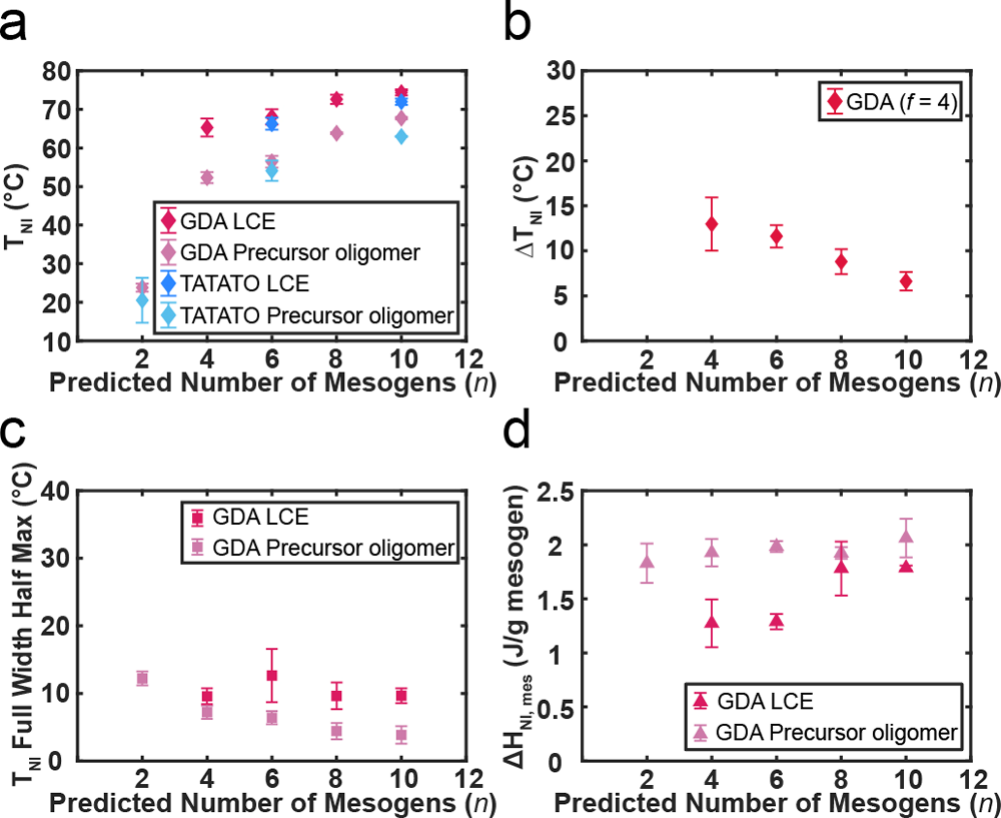
DSC measurements illustrating the impact of network strand molecular weight on the N–I transition. The length of the oligomers and network strands is denoted as *n*, the theoretical number of mesogens per chain. (a) *T*_NI_ of TATATO and GDA oligomers/LCEs as a function of *n* shows combined contributions of molecular weight and composition. (b) Δ*T*_NI_ of GDA LCEs as a function of *n*. (c) fwhm of GDA oligomer and LCE *T*_NI_ peaks shows the impact of compositional variance within a distribution of chain lengths for oligomers and the reduction ofquenched disorder for LCEs with increasing *n*. (d) Evolution of the normalized latent heat of the N–I transition with the molecular weight for GDA oligomers and LCEs. For all plots, the N–I transitions for *n* = 2 LCEs were too broad to accurately determine the thermal properties of the N–I transition.

In this study, changing the ratio of EDDT to C6M incurs a change in both the composition and degree of polymerization of the oligomers. Both the average fractional mesogen content of the chains and the amount of nonmesogenic cross-linker diluent added for a given LCE composition change with *n*. Ultimately, there is a net increase in the mesogen mass fraction incorporated into the oligomers and LCEs as *n* is increased. The simultaneous contributions from chain composition, mesogenic dilution by the cross-linker, and decrease in entropic differences between the nematic and isotropic states with molecular weight^55^ lead to the observed increase in *T*_NI_ with *n*. The Sm–N transition temperature also exhibits a comparable trend to *T*_NI_, likely derived from similar mechanisms (Figures S11a and S11d for GDA and TATATO, respectively). The glass transition temperature for the LCEs stays relatively constant irrespective of the network strand length (Figures S11b and S11e for GDA and TATATO, respectively).^52^

By examining Δ*T*_NI_ (Figure 5b), we probe the change in transition temperatures resulting from structural changes during the cross-linking reaction with a reduced degree of mesogenic dilution effects from the cross-linker. From rheological measurements, we find that longer network strands result in LCEs with lower *G*′ and thus a lower cross-link density and gel fraction (Figure S12). Therefore, Δ*T*_NI_ decreases with *n* mainly due to lowered cross-link density reducing the mesogen–backbone coupling. We note that *T*_NI_ and Δ*T*_NI_ of *n* = 2 LCEs were difficult to accurately determine due to the breadth of the transition peak and are thus not reported here.

While Δ*T*_NI_ between precursor oligomer and cross-linked states changes subtly with network strand length, more pronounced changes in both the breadth and enthalpy associated with the N–I transition are observed. The breadth of the N–I transition, or fwhm, is expected to increase upon cross-linking and is attributed to an increased degree of quenched disorder manifested as topological defects (bond disorder) and decreased first-order character (field disorder).^38,39^ As expected, the fwhm increases from the precursor oligomer to the LCE for GDA LCEs (Figure 5c, DSC traces shown in Figure S11c).

The fwhm of the *T*_NI_ peak generally decreases with the molecular weight for both the GDA precursor oligomers and LCEs (Figure 5c). The trend for the oligomers is more subtle than for the LCEs. For the precursor oligomers, a shallow decrease in fwhm is observed, attributed to a stronger dependence of *T*_NI_ on molecular weight for shorter oligomers than longer oligomers resulting in a broader distribution of *T*_NI_s for shorter chains and vice versa. For the LCEs, the fwhm of the *n* = 2 LCEs is not shown as the onset and end of the N–I transition are difficult to accurately determine. Nevertheless, the DSC scans for the GDA LCEs in Figure S11c (DSC scans for TATATO shown in Figure S11f) suggest a particularly large decrease in fwhm moving from *n* = 2 to *n* = 4, and a shallower decrease as *n* is further increased. This decrease in the breadth of the N–I transition of the LCEs is attributed to the reduction in cross-link density (and thus internal stresses).^38,39^

The latent heat is another key indicator of the first-order character of the N–I transition.^41,42,78^ To reduce the effects of mesogenic content, Δ*H*_NI,mes_ is plotted as a function of *n* for the oligomers and the LCEs in Figure 5d. The latent heat of the *n* = 2 GDA LCE was not quantifiable due to the convolution of the broad N–I transition peak and changes in heat capacity. Δ*H*_NI,mes_ of the GDA LCEs increases as the network strand length between cross-link junctions increases, appearing to plateau at longer *n*. This trend is attributed to a decrease in quenched field disorder with *n*, leading to a greater retention of first-order character.^38,39^

A larger N–I transition latent heat corresponds to an increased degree of first-order character that can be expected to manifest as a narrower phase transition with a smaller fwhm. The maximum achievable N–I transition latent heat for a given system of LCEs has an upper bound, determined by the latent heat of the precursor oligomers, which represents the zero cross-link density limit. An LCE for which the latent heat approaches that of its precursor oligomers would be expected to exhibit a transition featuring weakly first-order character corresponding to the theoretical limit for its composition. As a result, the rate of temperature-dependent actuation would also reach its theoretical limit within a given composition. For the precursor oligomers in Figure 5d, the normalized latent heat’s independence from oligomer molecular weight indicates that the upper bound for Δ*H*_NI,mes_ achievable for the corresponding LCEs is 1.95 ± 0.14 J g^–1^ mesogen, regardless of network strand length. This upper bound Δ*H*_NI,mes_ is lower than the reported value of Δ*H*_NI_ for pure C6M (2.47 J g^–1^).^83^ Controlling the distribution of latent heats that arise through the dispersity of the oligomer molecular weight distribution may be a promising route to narrow this gap and raise the ceiling for Δ*H*_NI,mes_. Further increasing this ceiling for latent heat would require synthesizing LCEs with mesogens that have a greater interaction strength than C6M.

## Conclusions

To specify how the network structure and mesogenic dilution collectively impact phase behavior, the roles of mesogen–backbone coupling, isotropic genesis, and composition in determining *T*_NI_ of polydomain LCEs were investigated by varying the cross-linker concentration in precursor oligomers and cross-linked LCEs while keeping the network strand length constant. Additionally, the impact of network strand length on the N–I transition was probed through varying the network strand length of LCEs with stoichiometrically balanced oligomer and cross-linker functional groups.

The increased mesogen–backbone coupling due to an increased degree of cross-linking was identified as the dominant factor in determining the magnitude of change in *T*_NI_ from the precursor oligomer to cross-linked LCE by reducing mesogenic dilution contributions through analysis of Δ*T*_NI_. However, an offset between Δ*T*_NI,max_ and the most cross-linked state associated with *G*′_max_ remains at higher cross-linker concentrations. Beyond the maximum degree of cross-linking, the increased degree of chain extension within the network leads to a decrease in mesogen–backbone coupling, in turn decreasing Δ*T*_NI_. Separately, when the network strand length of stoichiometric LCEs was increased, an increase in *T*_NI_ was observed. When the latent heat of the N–I transition was normalized by the mesogen weight fraction, it was shown to be independent of molecular weight, identifying an upper limit for the per gram mesogen latent heat achievable in this system of LCEs. Achieving a higher limit on the latent heat per gram (as might be desirable for elastocaloric materials) would require tuning of the oligomer molecular weight distribution or transitioning to mesogens with higher interaction strengths.

Through this study, the possibility of distinct network structures between LCEs synthesized with tri- and tetrafunctional cross-linkers was hypothesized, and TATATO networks were found to have significantly lower gel fractions than GDA networks with equivalent *G*′. While possible discrepancies in the network structure did not significantly impact the thermal properties in this study, the choice of cross-linker functionality is likely to have a substantial impact on the tensile mechanical properties and soft elasticity of LCEs, making it an important variable in the design of LCEs for energy dissipation applications. In addition, the impact of network strand length dispersity was not explored here. A more uniform network strand length is expected to sharpen the N–I transition peak and has the potential to open a new avenue for control over the speed of the transition, in turn having implications for actuation.

## Supplementary Material



## Data Availability

Extended data can be accessed at 10.34770/113f-6p86.

## Notes

Full reaction table detailing the composition of oligomers and LCEs; additional characterization of precursor oligomers and LCEs including thermal transition temperatures, transition peak fwhm, DSC scans, gel fraction measurements, GPC traces, and rheological measurements (PDF)

## †

Department of Chemical Engineering, Massachusetts Institute of Technology, Cambridge, Massachusetts 02139, United States (C.W.Z.)

## ‡

Stratingh Institute for Chemistry, University of Groningen, 9700 AB Groningen, The Netherlands (C.L.C.C.)
