# Supplementary material for: Competing Effects of Network Architecture and Composition on Polydomain Liquid Crystal Elastomers
Source: Macromolecules. 2026 Jan 22;59(3):1293–306. doi: 10.1021/acs.macromol.5c02541 (PMC12895520; doi:10.1021/acs.macromol.5c02541)
Supplement: Supplementary file 1 [file ma5c02541_si_001.pdf]

Supporting Information

Competing effects of network architecture and composition on polydomain liquid crystal elastomers

*David Taeyeun Yang,[1] Callie W. Zheng,[1] Chun Lam Clement Chan,[1] Shawn M. Maguire,[1] Emily C. Ostermann,[1] Emily C. Davidson[1] **

[1] Department of Chemical and Biological Engineering, Princeton University, Princeton NJ 08544

a

Calculated Crosslinker Equivalents	Amount of material added (g)					
	RM82	EDDT	BHT	IG369	GDA	TEA
0					0.0000	
0.022					0.0166	
0.044					0.0333	
0.066					0.0499	
0.083	2.0000	0.6323	0.0526	0.0395	0.0630	0.0263
0.09					0.0680	
0.093					0.0703	
0.11					0.0832	
0.132					0.0998	

b

Calculated Crosslinker Equivalents	Amount of material added (g)					
	RM82	EDDT	BHT	IG369	TATATO	TEA
0					0.0000	
0.022					0.0163	
0.044					0.0326	
0.066					0.0489	
0.088	2.0000	0.6323	0.0526	0.0395	0.0652	0.0263
0.111					0.0823	
0.120					0.0889	
0.132					0.0978	
0.154					0.1141	

c

Predicted Number of Mesogens (n)	Amount of material added (g)					
	RM82	EDDT	BHT	IG369	TATATO	TEA
2		0.8129	0.0563	0.0422	0.2470	0.0281
6	2.0000	0.6323	0.0526	0.0395	0.0823	0.0263
10		0.5961	0.0519	0.0389	0.0519	0.0260

d

Predicted Number of Mesogens (n)	Amount of material added (g)					
	RM82	EDDT	BHT	IG369	TATATO	TEA
2		0.8129	0.0563	0.0422	0.2470	0.0281
6	2.0000	0.6323	0.0526	0.0395	0.0823	0.0263
10		0.5961	0.0519	0.0389	0.0519	0.0260

Table S1. Reaction formulation tables for (a), (b) LCEs with varied TATATO and GDA content, respectively, and (c), (d) GDA and TATATO LCEs with varied network strand lengths, respectively. Columns with one value are added in the same amount throughout.

a

TATATO Precursor Oligomer

Crosslinker Concentration (Eq)	Average T_{NI} (°C)	T_{NI} Standard Deviation (°C)	NI Latent Heat (J/g)	NI Latent Heat Standard Deviation (J/g)
0	63.1667	1.0451	1.4409	0.1305
0.022	62.9033	0.7596	1.4809	0.0387
0.044	59.7000	0.7950	1.4659	0.0472
0.066	58.8633	0.9815	1.4358	0.1000
0.088	55.9533	1.4924	1.2827	0.0321
0.11	54.0867	2.6658	1.3801	0.0505
0.12	52.0800	1.4118	1.3486	0.0862
0.132	51.3333	0.9950	1.3162	0.0867
0.154	51.2100	1.2813	1.2758	0.0883

b

TATATO LCE

Crosslinker Concentration (Eq)	Average T_{NI} (°C)	T_{NI} Standard Deviation (°C)	NI Latent Heat (J/g)	NI Latent Heat Standard Deviation (J/g)
0	66.0833	1.1201	1.3710	0.0798
0.022	67.0333	0.6753	1.3188	0.0674
0.044	68.2867	0.7974	1.2564	0.0516
0.066	68.0367	2.2082	1.1167	0.0885
0.088	67.6800	0.4807	1.0307	0.0808
0.11	66.2623	1.5569	0.9498	0.0925
0.12	61.9200	0.9619	1.0023	0.0848
0.132	60.1433	0.1159	1.0002	0.1019
0.154	60.3267	0.5024	0.9888	0.0421

c

Crosslinker Concentration (Eq)	Average G' (Pa)	G' Standard Deviation (Pa)
0	3548.5000	677.0166
0.022	16323.6667	3084.5759
0.044	49557.6667	7147.3733
0.066	89748.0000	2201.4443
0.088	115773.3333	4743.0089
0.11	170676.6667	8345.1802
0.12	218770.0000	3824.5392
0.132	214343.3333	2707.7912
0.154	151340.0000	7232.5583

Table S2. T_{NI} and ΔH_{NI} and corresponding standard deviations for TATATO oligomers (a) and LCEs (b) of different crosslinker concentrations. (c) Average G' for TATATO LCEs and standard deviations.

a

GDA Precursor Oligomer

Crosslinker Concentration (Eq)	Average T_{NI} (°C)	T_{NI} Standard Deviation (°C)	NI Latent Heat (J/g)	NI Latent Heat Standard Deviation (J/g)
0	63.1667	1.0451	1.4409	0.1305
0.022	61.4700	0.9781	1.3900	0.0403
0.044	58.9433	1.5128	1.2057	0.0414
0.066	58.0367	1.8103	1.2374	0.0828
0.083	56.4200	1.5001	1.2112	0.0586
0.09	56.7867	2.2601	1.1661	0.0621
0.093	57.5067	1.0600	1.1983	0.0641
0.11	55.9867	2.3236	1.1860	0.0455
0.132	57.2033	2.5408	1.0773	0.1926

b

GDA LCE

Crosslinker Concentration (Eq)	Average T_{NI} (°C)	T_{NI} Standard Deviation (°C)	NI Latent Heat (J/g)	NI Latent Heat Standard Deviation (J/g)
0	66.0833	1.1201	1.3710	0.0798
0.022	65.5367	1.7721	1.3009	0.0508
0.044	66.5000	0.4095	1.2733	0.0527
0.066	70.8000	0.8561	1.0836	0.0801
0.083	68.0433	1.9848	0.9252	0.0511
0.09	70.6900	0.3863	1.0254	0.0344
0.093	65.4033	0.9478	0.9667	0.0776
0.11	63.4933	0.8334	0.9410	0.1138
0.132	61.6967	1.3916	0.8574	0.1333

c

Crosslinker Concentration (Eq)	Average G' (Pa)	G' Standard Deviation (Pa)
0	3548.5000	677.0166
0.022	21858.6667	4604.3191
0.044	98863.0000	1894.2595
0.066	117603.3333	6798.1566
0.083	161006.6667	18139.3752
0.09	175980.0000	8290.0724
0.093	167306.6667	1909.5898
0.11	206383.3333	4371.8913
0.132	187856.6667	22159.7954

Table S3. T_{NI} and ΔH_{NI} and corresponding standard deviations for GDA oligomers (a) and LCEs (b) of different crosslinker concentrations. (c) Average G’ for GDA LCEs and standard deviations.

a

GDA Precursor Oligomer

n	Average T_{NI} (°C)	T_{NI} Standard Deviation (°C)	NI Latent Heat (J/g)	NI Latent Heat Standard Deviation (J/g)
2	23.8033	1.0538	0.1174	12.2222
4	52.2967	1.4244	0.0885	7.2100
6	56.4200	1.5001	0.0362	6.4000
8	63.8067	0.2259	0.0397	4.4233
10	67.7233	0.3009	0.1318	3.8533

b

GDA LCE

n	Average T_{NI} (°C)	T_{NI} Standard Deviation (°C)	NI Latent Heat (J/g)	NI Latent Heat Standard Deviation (J/g)
2	33.3233	2.9843	0.2140	33.8433
4	65.2795	2.5031	0.1542	9.5739
6	68.0433	1.9848	0.0511	12.6500
8	72.6100	1.1805	0.1820	9.6267
10	74.3367	0.7467	0.0159	9.6667

c

n	Average G' (Pa)	G' Standard Deviation (Pa)
2	543076.6667	10821.3508
4	286093.3333	25431.3593
6	161006.6667	18139.3752
8	101512.6667	4861.7241
10	56334.0000	12387.0847

Table S4. T_{NI} and ΔH_{NI} and corresponding standard deviations for GDA oligomers (a) and LCEs (b) of different predicted mesogens per chain n. (c) Average G' for LCEs and standard deviations.

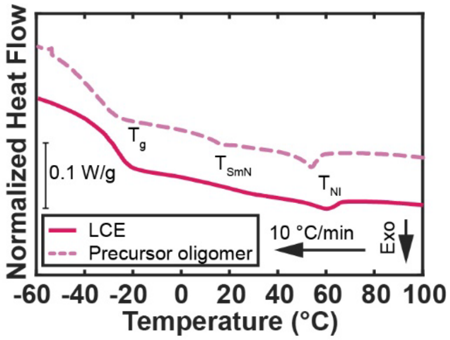

Figure S1. DSC third cooling scan for n = 6 GDA LCE and precursor oligomer.

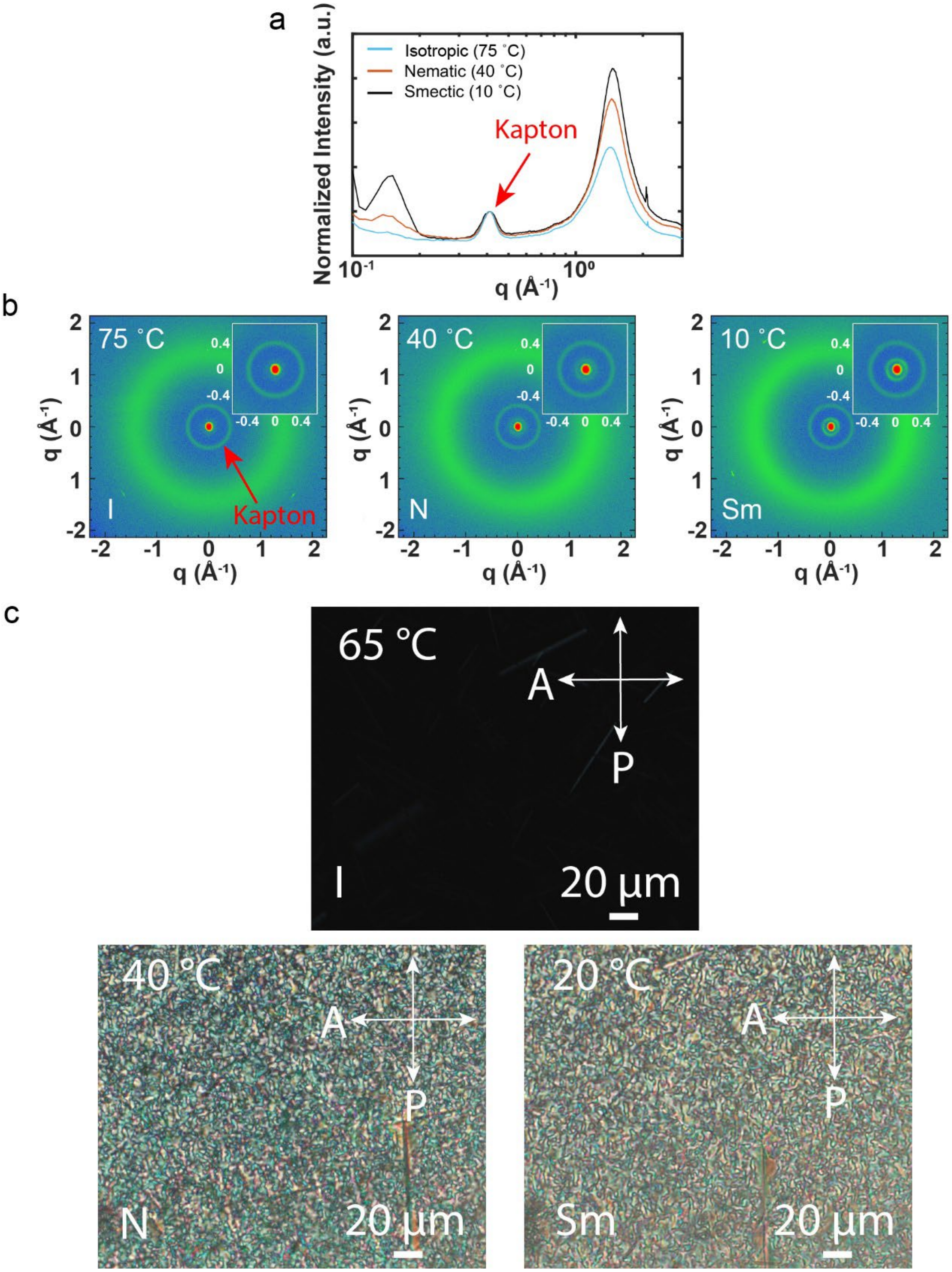

Figure S2. 1D and 2D wide-angle x-ray scattering (WAXS) data and polarized optical microscopy (POM) images for *n* = 6 GDA oligomer with 0.083 eq GDA. (a) 1D scattering pattern showing growth of smectic peak at ~0.14 Å^{-1}. The peak at ~0.4 Å^{-1} corresponds to Kapton used to make the measurement cells. (b) 2D WAXS scattering patterns in the isotropic, nematic, and smectic phase for the same oligomer. Insets: Close-up view of the evolution of the inner halo. (c) POM images of the same oligomer in the isotropic, nematic, and smectic phase. POM samples were cooled at a rate of 0.1 °C/min.

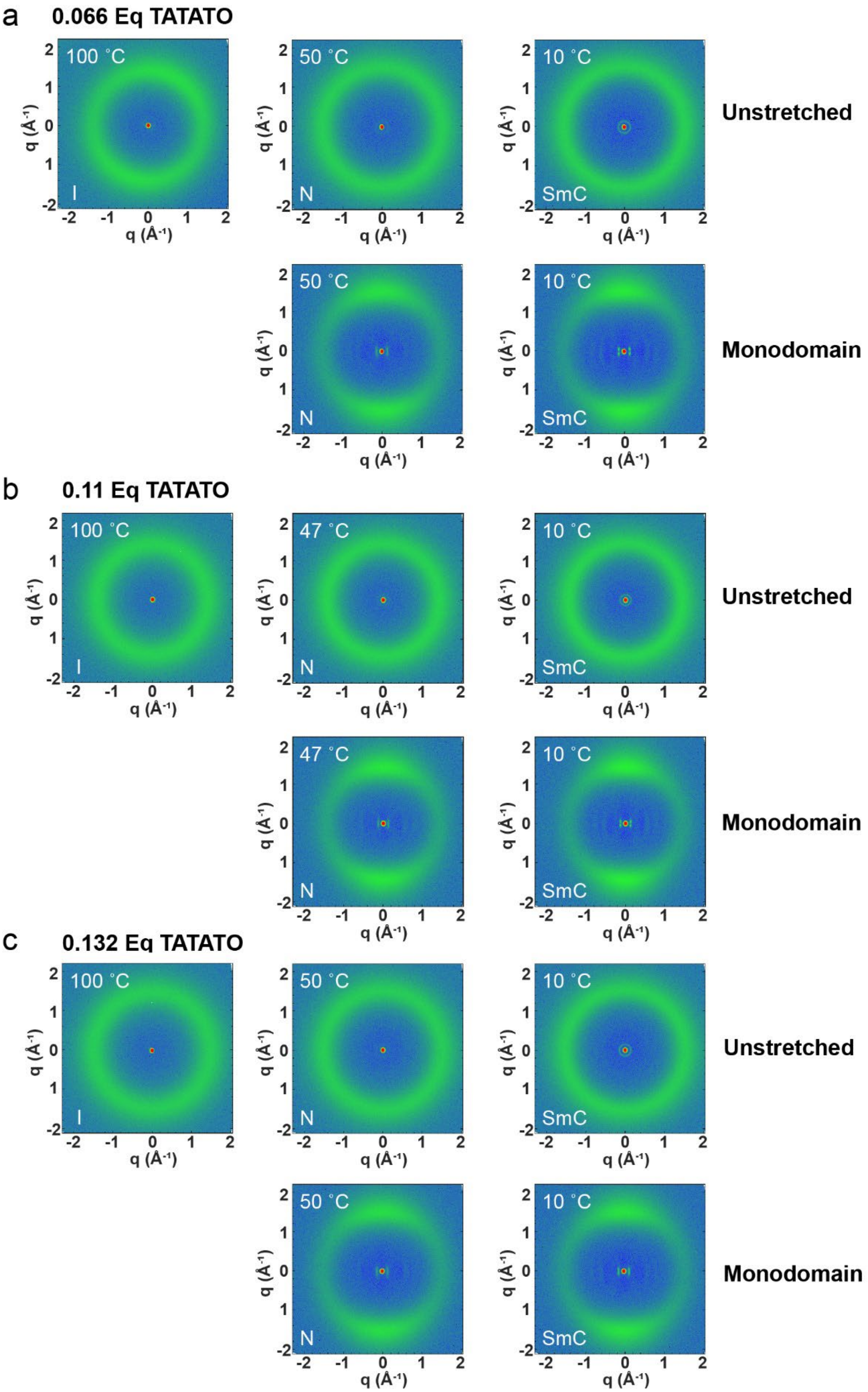
a
0.066 Eq TATATO
100 °C
I
50 °C
N
10 °C
SmC
Unstretched
50 °C
N
10 °C
SmC
Monodomain
b
0.11 Eq TATATO
100 °C
I
47 °C
N
10 °C
SmC
Unstretched
47 °C
N
10 °C
SmC
Monodomain
c
0.132 Eq TATATO
100 °C
I
50 °C
N
10 °C
SmC
Unstretched
50 °C
N
10 °C
SmC
Monodomain
q (Å⁻¹)

Figure S3. 2D WAXS scattering patterns for TATATO LCEs with varied TATATO loadings. Samples were taken in the isotropic, nematic, and smectic phase. Nematic and smectic samples were measured in both the stretched (150% engineering strain) and unstretched states.

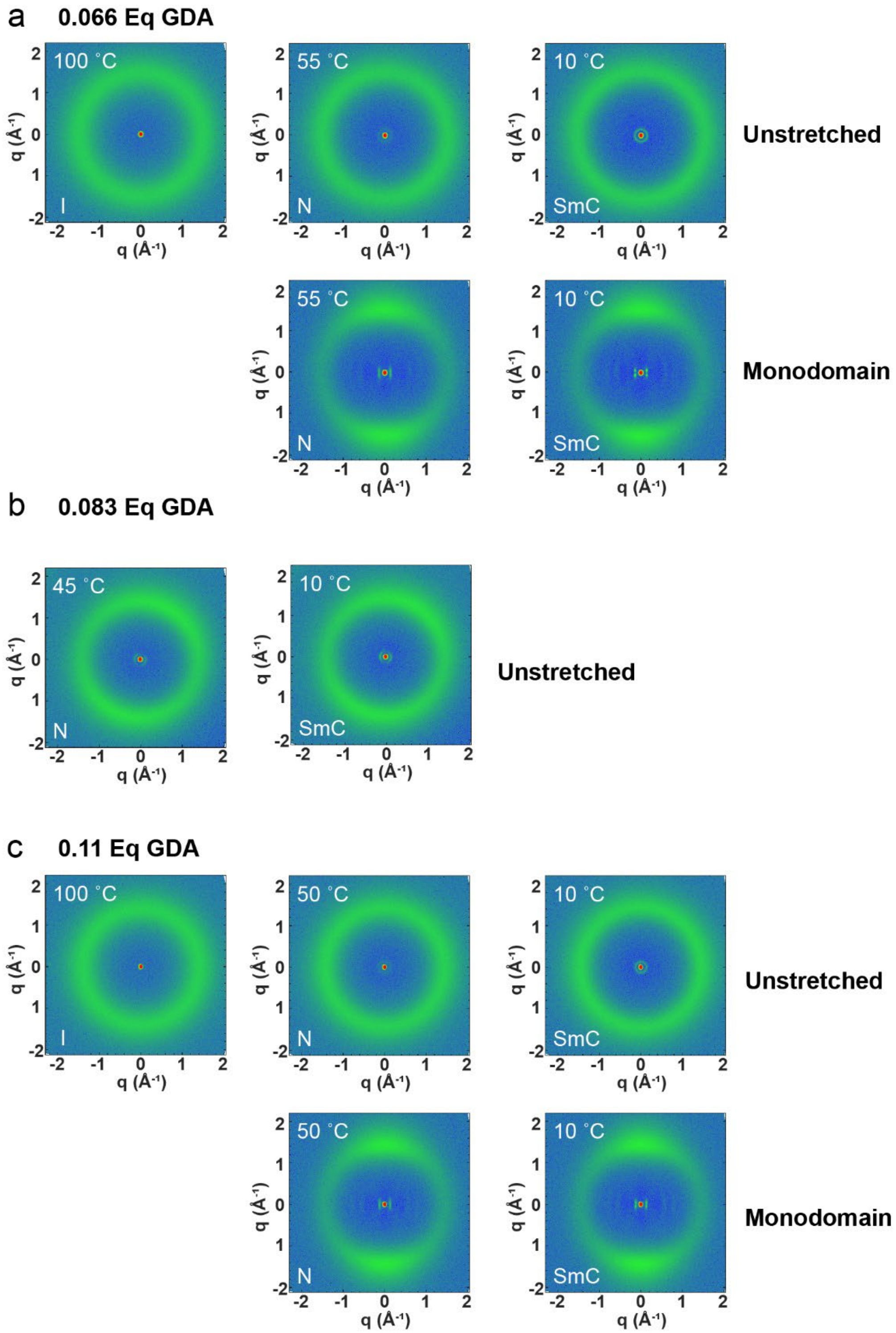

Figure S4. 2D WAXS scattering patterns for GDA LCEs with varied GDA loadings. Samples were taken in the isotropic, nematic, and smectic phase. Nematic and smectic samples were measured in both the stretched (150% engineering strain) and unstretched states.

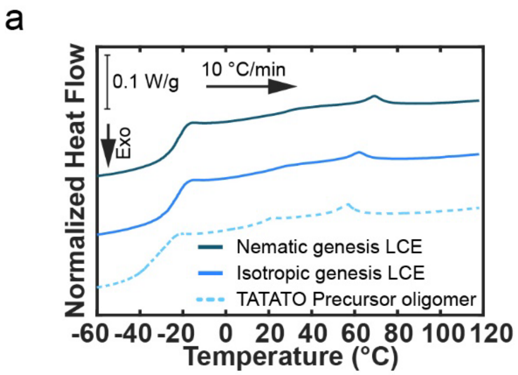

Figure S5. DSC heating scans for precursor oligomers, isotropic genesis LCEs, and nematic genesis LCEs for (a) TATATO, (b) GDA.

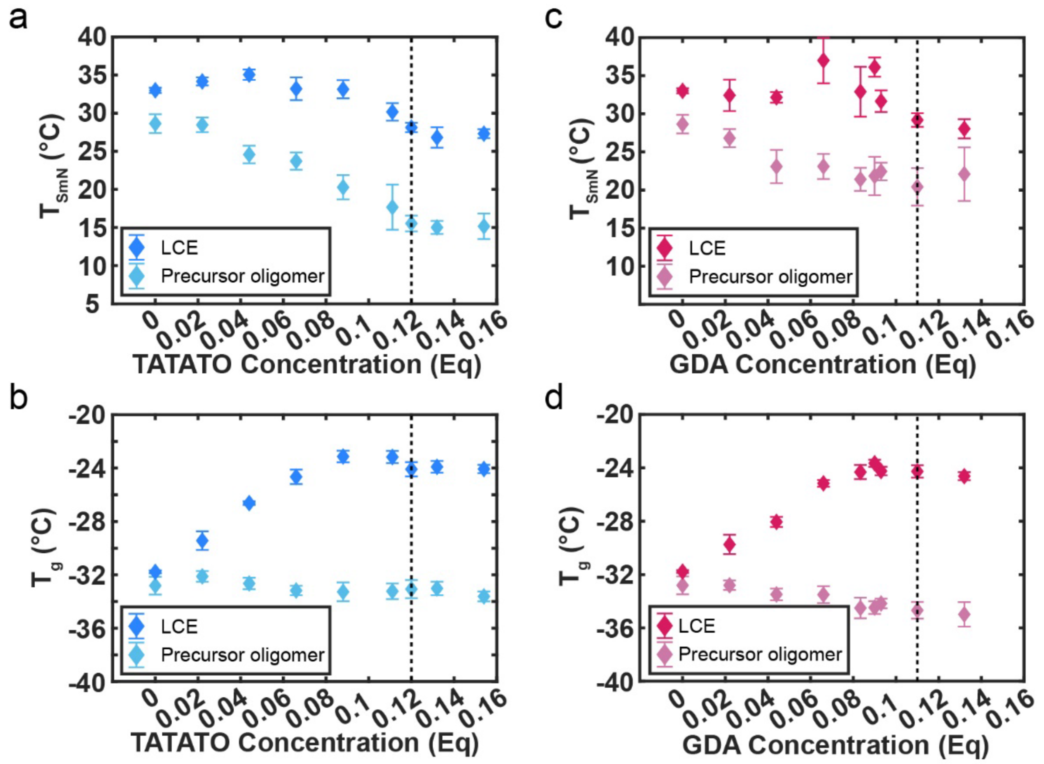

Figure S6. Smectic-to-nematic transition and glass transition temperatures for (a), (b) TATATO and (c), (d) GDA precursor oligomers and LCEs as a function of crosslinker concentration. The black dotted line represents the composition corresponding to the maximum G'.

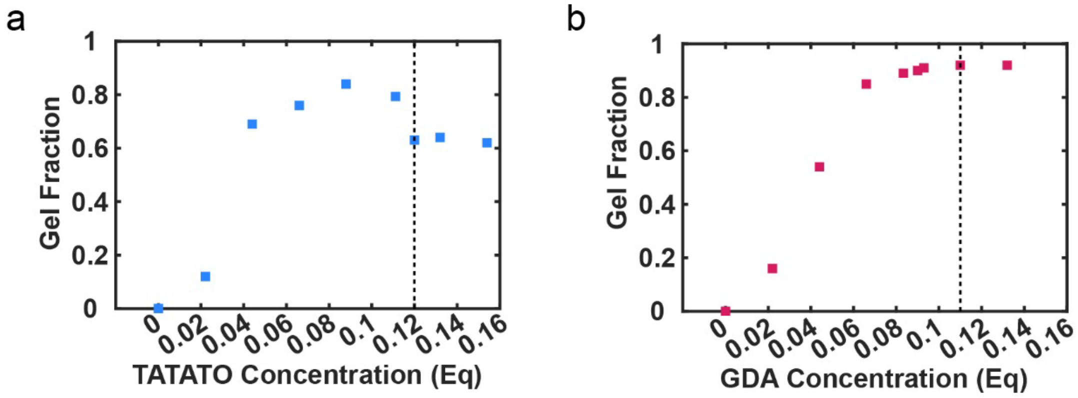

Figure S7. Gel fractions for (a) TATATO and (b) GDA LCEs as a function of crosslinker concentration. The black dotted line represents the composition corresponding to the maximum G'.

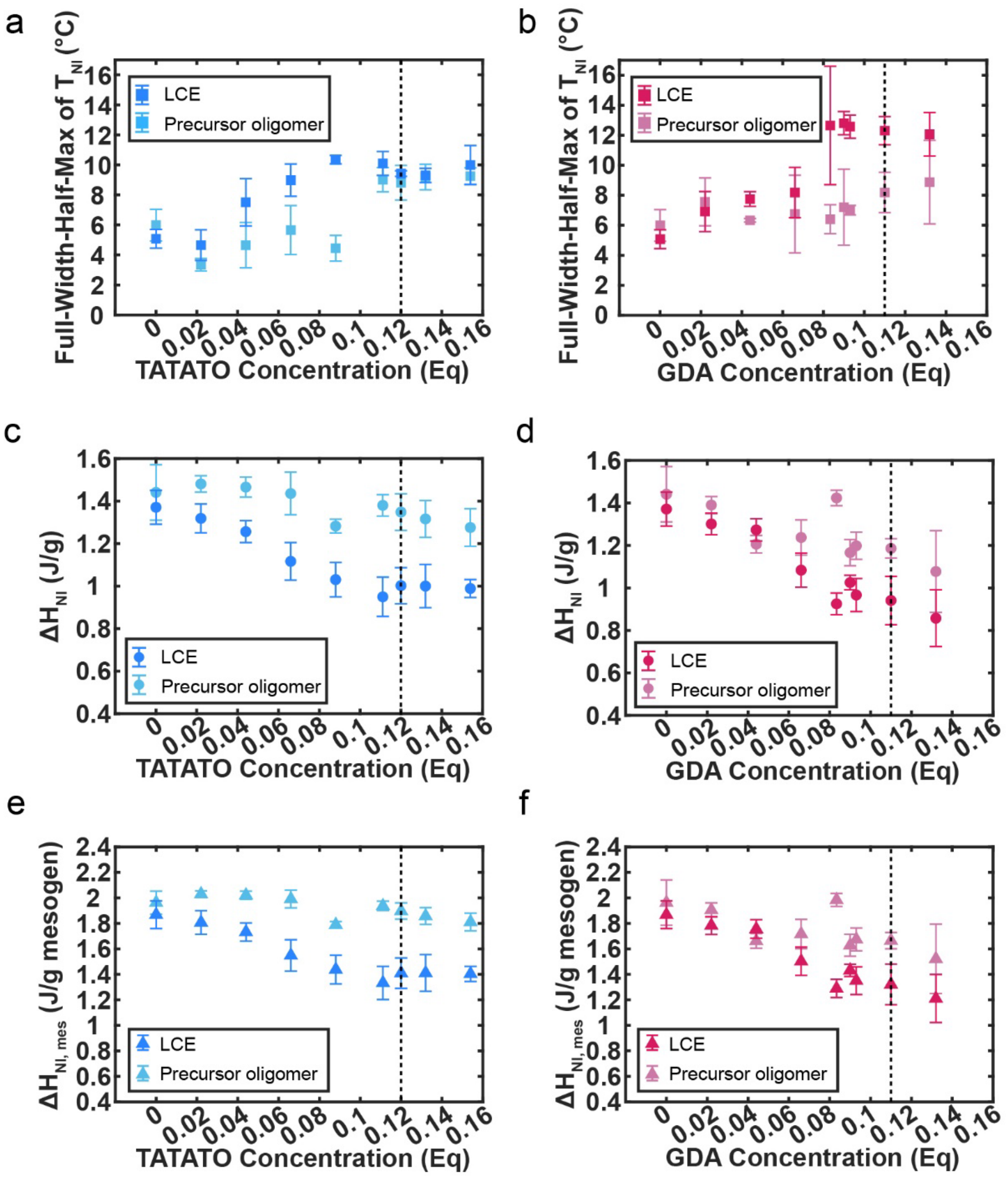

Figure S8. Full-width half-max of nematic-to-isotropic transition ((a), (b)), ΔH_{NI} ((c), (d)), and $\Delta H_{NI,\,mes}$ ((e), (f)) as a function of crosslinker concentration for TATATO and GDA LCEs and precursor oligomers. The black dotted line represents the composition corresponding to the maximum G'. as a function of crosslinker concentration.

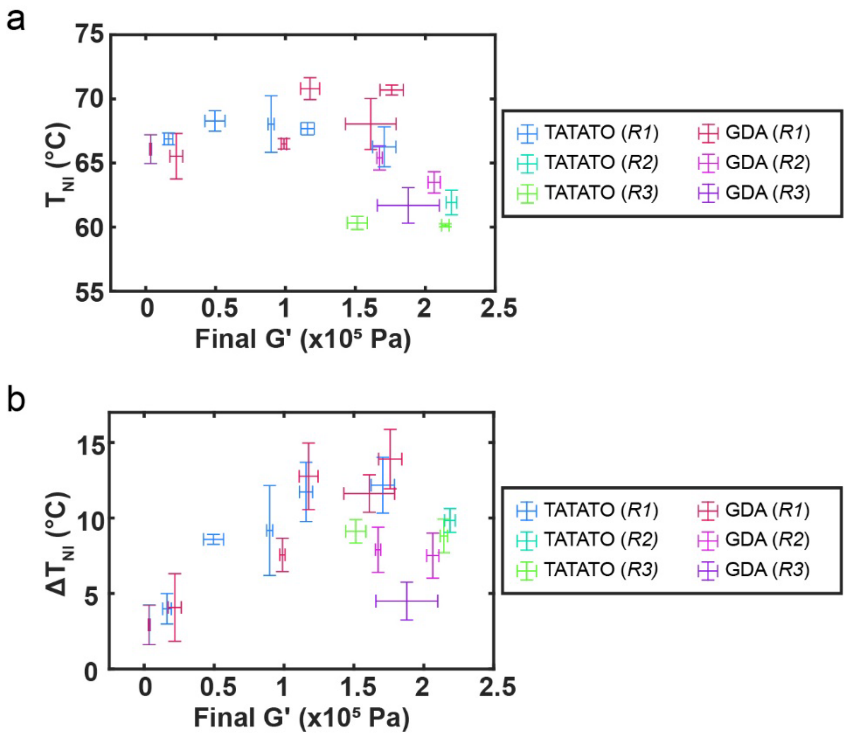

Figure S9. (a) LCE T_{NI} plotted against G' and (b) ΔT_{NI} plotted against G'. LCEs with crosslinker concentrations in R1, R2, and R3 are colored in light blue, teal, and light green, respectively for TATATO and red, pink, and purple, respectively, for GDA.

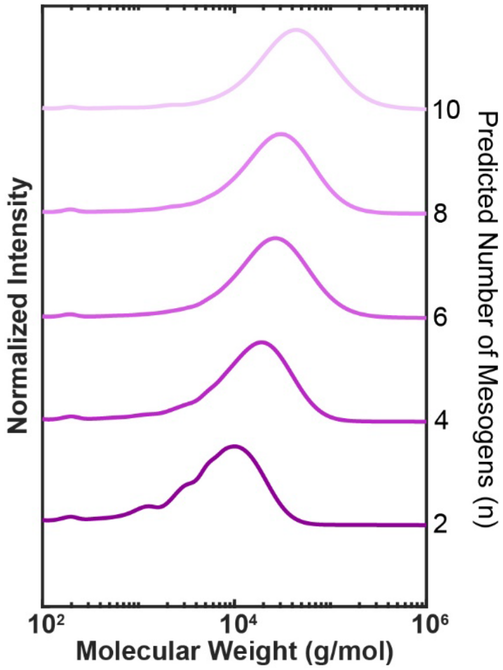

Figure S10. (a) Molecular weight distributions of LC polymers of varied predicted number of mesogens per chain.

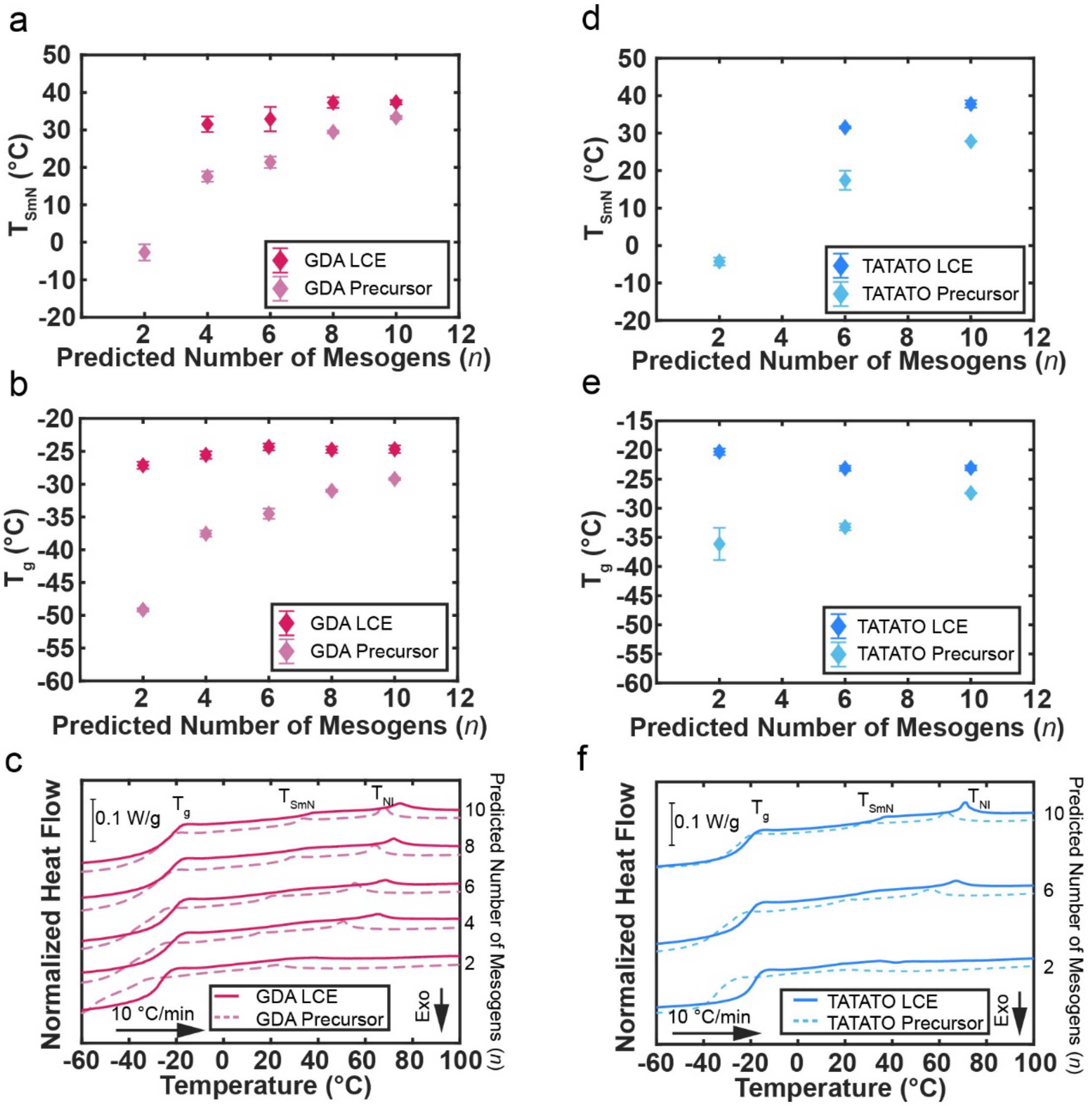

Figure S11. Smectic-to-nematic transition temperatures, glass transition temperatures, and DSC traces for (a), (b), (c) GDA and (d), (e), (f) TATATO precursor oligomers and LCEs as a function of predicted number of mesogens per oligomer/network strand. The *Sm-N* transition for the n = 2 LCEs was not observed in the DSC measurements.

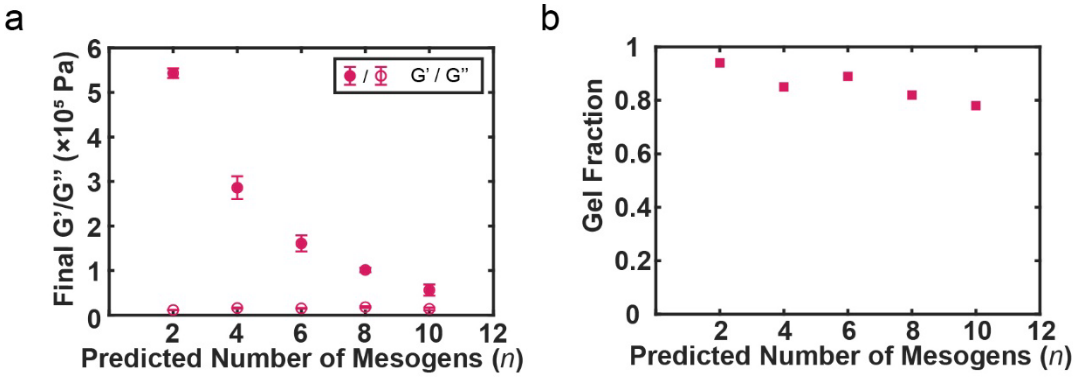

Figure S12. (a) Storage (G') and loss (G'') moduli and (b) gel fractions of GDA LCEs crosslinked with different network strand lengths.
